# Influence of genetic diversity, drought stress and rhizobial symbiosis on the nutritional quality of common vetch (*Vicia sativa* L.) grain

**DOI:** 10.1002/jsfa.70410

**Published:** 2026-03-02

**Authors:** María Isabel López‐Román, Lucía De la Rosa, Cristina Castaño‐Herrero, Maria Teresa Marcos‐Prado, Elena Ramírez‐Parra

**Affiliations:** ^1^ Centro de Biotecnología y Genómica de Plantas, (CBGP, UPM‐INIA/CSIC) Instituto Nacional de Investigación y Tecnología Agraria y Alimentaria, Consejo Superior de Investigaciones Científicas Universidad Politécnica de Madrid Pozuelo de Alarcón Spain; ^2^ Centro de Recursos Fitogenéticos (CRF‐INIA/CSIC) Instituto Nacional de Investigación y Tecnología Agraria y Alimentaria, Consejo Superior de Investigaciones Científicas Alcalá de Henares Spain

**Keywords:** drought stress, legume, common vetch, symbiosis, genetic resources

## Abstract

**BACKGROUND:**

Legumes are the primary source of plant protein in both human and livestock diets and, therefore, play an essential role in nutrition. Common vetch (*Vicia sativa* L.) is a grain legume widely used in animal feed. Its nutritional properties, particularly its high protein content, make it an adequate component to enrich feedstuffs. Common vetch, like other legumes, is essential in sustainable agriculture systems in mitigating soil degradation and reducing the need for chemical fertilizers, due to its ability to fix atmospheric nitrogen, but it is increasingly being affected by drought – one of the main environmental factors that reduces its production. The genetic diversity among different varieties and environmental conditions may significantly impact the productivity and nutritional composition of legume grains.

**RESULTS:**

In this study, we explore the effect of intra‐species genetic diversity on protein levels, carbohydrates, minerals and the composition of specific nutrients, antioxidants and antinutritional factors. We have also analyzed the impact of drought stress and rhizobial symbiosis on the nutritional quality of vetch grain. Our results indicate that there are specific alterations in the selective enrichment or depletion of certain nutrients and ANFs among the diverse *V. sativa* accessions that have been analyzed but, interestingly, there are also differences in grain composition under different treatments, such as water deprivation, or in plants that have established rhizobial symbiosis.

**CONCLUSION:**

Our findings suggest that the combined effect of genotype and environment, such as drought or symbiosis, plays an important role in the nutritional composition of the grain legume *V. sativa*. © 2026 The Author(s). *Journal of the Science of Food and Agriculture* published by John Wiley & Sons Ltd on behalf of Society of Chemical Industry.

## INTRODUCTION

After cereals, legumes (*Fabaceae* family) are the second most relevant family of crops. They have significant economic and ecological importance, providing over a third of the global population's protein consumption for humans and livestock.^1^ Moreover, the legume family is considered unique for its ability to establish symbiosis with nitrogen‐fixing bacteria. This biological nitrogen fixation (BNF) is a substantial source of nitrogen for agricultural soils and plays a key role in improving soil fertility and reducing the need for synthetic fertilizers, minimizing the cost for farmers and lowering the associated pollution levels, contributing to sustainable agriculture and circular economy systems.^2^, ^3^

Drought is one of the main threats to crop production, especially in rainfed legumes, such as common vetch.^4^ Common vetch (*Vicia sativa* L.) is an annual legume with economic relevance worldwide, commonly used as animal feed, both as forage and as grain legume. Common vetch also has great environmental value due to its use in rotation practices and as a cover crop, helping to regenerate and improve soil quality.^5^ The Mediterranean Basin is the region of origin and primary diversification of this species, according to archaeological evidence.^6^, ^7^ However, nowadays vetch can be found all over the world. Based on average last decade data from FAOSTAT, the world vetch production was 796.939 tons per year and the top producers were Ethiopia, the Russian Federation, Mexico and Spain.^5^, ^8^

Several recent studies have examined the nutritional value of common vetch as a dietary component for animal feed.^5^, ^9^, ^10^ Its exceptional digestibility, elevated energy value, high protein and mineral content make it an ideal component to enrich feedstuffs.^9^, ^11^ Regardless of the well‐known lack of sulfur‐containing amino acids (methionine and cysteine), the major conclusions of these studies support the potential of common vetch grain as a rich source of nutrients with a lower economic cost than other options. However, in addition to beneficial nutrients, *V. sativa* seeds contain antinutritional factors (ANFs), such as condensed tannins and other phenolic secondary metabolites, which reduce palatability and can limit nutrient absorption and utilization. Previous analyses of the nutritional content of the vetch grain revealed significant variations across Eastern European genotypes from different geographic origins in terms of ANFs, protein content, fatty acid composition and mineral composition.^9^, ^12^, ^13^, ^14^ Despite the limited scope of these investigations, the results support the utilization of the diversity of genetic resources that exist in *V. sativa* gene banks as a tool for exploring the nutritional properties of different genotypes for breeding purposes.^9^, ^12^, ^13^, ^14^ Gene banks are valuable sources for the diversity conservation of the genetic resources of different common vetch genotypes. The gene bank of the National Spanish Plant Genetic Resources Center belongs to INIA‐CSIC and conserves one of the largest common vetch world collections, with more than 1000 accessions, including landraces, wild populations and commercial cultivars of both Spanish and international origin. During recent years, numerous studies have been carried out to characterize the variability of this common vetch collection by genotyping and by evaluating agronomic parameters, mainly associated with production, phenology and drought tolerance.^15^ However, there is barely any characterization of the grain nutritional properties of different genotypes. Grain composition in food legumes may be affected by abiotic stresses, including drought.^16^ Thus, alterations in total protein and lipid content and the concentration of Fe, P and Zn have been reported in a small number of bean varieties under drought conditions.^17^ Regarding the impact of inoculation on nutritional composition, few studies address the influence of rhizobial symbiosis on the nutritional composition of legumes. Recently, it has been described that inoculation of legumes with rhizobia not only affects nitrogen uptake by plants but also the absorption of other elements (Mg, P, K and Ca) in both faba bean and soybean leaves and causes alterations of P, K, Fe and Mn concentrations in seeds.^18^

From previous genotypic analyses developed in our laboratory, we have selected a group of 19 accessions that include the main representative genetic and phenotypic diversity of the complete common vetch collection.^15^, ^19^ Hence, this study aims, first, to analyze and explore the variability in the nutritional composition of different common vetch genotypes. Subsequently, we analyzed the impact of drought stress and rhizobial symbiosis on the nutritional composition of vetch seeds. This study aims to find correlations between genetic diversity, agro‐morphological characterization data and nutritional component diversity in the analyzed accessions.

## MATERIALS AND METHODS

### Plant materials and treatments

The description and passport data of the 19 *V. sativa* accessions analyzed in this work are described in Supporting Information, Table S1. Complete data are available at the CRF (https://eurisco.ipk‐gatersleben.de/apex/eurisco/r/eurisco/home). These accessions were selected, based on previous analyses performed in our laboratory, to represent the main genetic and phenotypic diversity of the complete common vetch collection along with three commercial varieties: Aitana, Senda and Verdor.^15^, ^19^ For nutritional composition analysis, *V. sativa* seeds of each accession were germinated and grown in a greenhouse with controlled conditions (photoperiod: 16 h light/8 h dark, at 22 °C). Plants were grown under the indicated controlled conditions with the specified treatments until seed production. Grain was desiccated and stored until sample extraction.

For assays of rhizobial symbiosis, the seeds were surface sterilized (sequentially by 96% ethanol and 12% bleach) and germinated on 1% agar plates. Varieties BGE025608, BGE014901 and Senda were used in this treatment. Inoculation experiments were performed in sterilized Leonard jars with vermiculite substrate and an N‐free hydroponic nutrient solution.^20^ Seedlings were infected with 1 mL of the early stationary rhizobial culture. *Rhizobium leguminosarum* bv. *viciae* (Rlv) strain V31 was employed as the inoculant.^19^, ^21^ Ten days after sowing, N‐fed plants used as non‐inoculated control were cultivated in the same nutrient solution enriched with 10 mmol L^−1^ NO_3_NH_4_ ^21^ and both treatments were transferred to sterilized soil 2 weeks after inoculation and grown until grain production, under controlled greenhouse conditions (photoperiod: 16 h light/8 h dark, at 22 °C). Nodule formation and nitrogenase activity were tested to assess effective nodulation. Drought treatments were applied to 4‐week‐old plants (accessions BGE022207, BGE016970 and Senda) by water deprivation for 4 weeks, until grain production, under controlled greenhouse conditions (photoperiod: 16 h light/8 h dark, at 22 °C). The experiment timeline of both treatments is shown in Supporting Information, Fig. S1.

### Field characterization of agro‐morphological traits

The evaluation of agro‐morphological traits of *V. sativa* accessions was carried out during the 2022–2023 growing season at ‘Finca La Canaleja’, Alcalá de Henares, Madrid (602 m above sea level; 40° 30′ 54″ N, 03° 18′ 42″ W; average temperature 13.7 °C and average annual rainfall 420 mm; soil characterization: calcium alfisol, loam, saturation of bases (100%), moderately alkaline (pH 8.4) and organic carbon (0.6%). The agro‐morphological traits studied of plants, flowers, pods, fruits, seeds and phenology were selected following the recommendation of IBPGR (now Alliance Biodiversity & CIAT) for species of *Vicia* genus and the International Union for the Protection of New Varieties of Plants (UPOV) guidelines for *V. sativa* L.

### Sample extraction and determination of antioxidants and ANFs

Seventy‐five milligrams of dry vetch seeds were mortar ground and extracted with 1.5 mL methanol–HCl (99:1) for 2 h with agitation (130 × g) at 25 °C under dark conditions. Methanolic extracts were centrifuged, and the supernatant was stored in the dark at −20 °C until analysis.^22^

Anthocyanin determination was directly performed spectrophotometrically over methanolic extract of vetch flour at 530 nm and corrected by 650 nm,^23^ using a SPECTROstar Nano spectrophotometer (BMG Labtech, Ortenberg, Germany). At least six sample replicas were measured.

Total phenolic content (TPC) of vetch seed extracts was determined as described previously,^24^ with modifications. Reactions were performed in a 96‐well plate (200 μL of final volume). Briefly, 20 μL methanolic extract was mixed with 50 μL Folin–Ciocâlteu reagent solution (0.25 mol L^−1^) and 80 μL water. After 8 min, the reaction was neutralized with 50 μL of 20% sodium carbonate solution. The reaction was incubated at 25 °C for 2 h under dark conditions, and absorbance was measured at 765 nm using a SPECTROstar‐Nano spectrophotometer (BMG Labtech). TPC was calculated using a standard curve of gallic acid (0–300 μmol L^−1^). Values were presented as milligrams of gallic acid equivalents (GAE) per gram of seed flour dry weight. At least four sample replicates were measured.

Total flavonoid content (TFC) of vetch seed extracts was quantified as reported previously,^25^ with some modifications. Reactions were performed in a 96‐well plate (200 μL of final volume). Fifty microliters of methanolic extract were mixed with 20 μL 2‐aminoethyldiphenyl borate solution (10 mg m L^−1^) and 130 μL water. The reaction was incubated at 25 °C for 10 min under dark conditions, and absorbance was measured at 404 nm using a SPECTROstar Nano spectrophotometer (BMG Labtech). TFC was calculated using a standard curve of rutin (0–250 μg m L^−1^). Values were presented as milligrams of rutin equivalents (RE) per gram of seed flour dry weight. At least six sample replicates were measured.

Total tannin content (TTC) of vetch seed extracts was determined as reported previously,^24^ with modifications. Reactions were done in a 96‐well plate (120 μL of final volume). Briefly, 20 μL methanolic extract was mixed with 100 μL reactive vanillin (vanillin 1% and HCl 8%; 1:1, v/v). The reaction was incubated at 25 °C for 2 h under dark conditions, and absorbance was measured at 500 nm using a SPECTROstar Nano spectrophotometer (BMG Labtech). TTC was calculated using a standard curve of catechin (0–1 mg m L^−1^). Values were presented as milligrams of catechin equivalents (CE) per gram of seed flour dry weight. At least six sample replicas were measured.

The 2,2‐diphenyl‐1‐picrylhydrazyl (DPPH) assay of vetch seed extracts for the evaluation of radical scavenging activity was performed as described previously,^25^ with some modifications. Briefly, 4 μL of each methanolic extract was mixed with 180 μL of 0.1 mmol L^−1^ DPPH radical solution and 16 μL water. The reaction was incubated at 25 °C for 25 min under dark conditions, and absorbance was measured at 515 nm using a SPECTROstar Nano spectrophotometer (BMG Labtech). DPPH activity was calculated using a standard curve of Trolox (0–20 mg m L^−1^). Values were presented as milligrams of Trolox equivalents (TE) per gram of seed flour dry weight. At least six sample replicates were measured.

### Total sugar and starch quantification

Soluble sugars were quantified as previously described by using the anthrone reagent, as described by Yemm and Willis^26^ and by Del Pozo and Ramirez‐Parra.^27^

### Metabolomic analyses, ionomic analyses, protein determination and carbon/nitrogen content

The analyses of primary metabolites (organic acids, sugars and amino acid content) were determined by proton nuclear magnetic resonance (^1^H‐NMR) at the Metabolomics Platform of CEBAS (CSIC, Spain). For the elemental analyses of C/N, grain flour was oven‐dried (65 °C) for 7 days and total nitrogen and carbon contents were estimated by the Dumas method using a TruSpec C/N analyzer (LECO Corp., St Joseph, MI, USA) at the Ionomics Platform of CEBAS. For the elemental analyses of minerals, grain flour was weighed, and total nitrogen and carbon contents were estimated by inductively coupled plasma–optical emission spectrometry (ICP‐OES) using a Thermo iCAP 6500 Duo ICP‐AES spectrometer (Thermo Fisher Scientific, Waltham, MA, USA) at the Ionomics Platform of CEBAS.

Crude protein was calculated as N × 6.25, where N is the nitrogen obtained by mineralization through the Dumas method. Soluble protein extracts were prepared as follows: 100 mg grain was mortar‐ground and extracted with 1 mL buffer (100 mmol L^−1^ Tris–HCl pH 8.0; 300 mmol L^−1^ NaCl; 0.1% (w/v) sodium dodecyl sulfate (SDS); 1 mmol L^−1^ ethylenediaminetetraacetic acid (EDTA); 0.1% (v/v) β‐mercaptoethanol; 0.2% (v/v) NP40 and protein inhibitors (Sigma‐Aldrich, St Louis, MO, USA)). Extracts were sonicated three times for 30 s each time (one cycle, 60 amplitude; Sonicator Hielscher UP100H, Hielscher Ultrasonics GmbH, Teltow, Germany) and incubated at 4 °C with constant rocking overnight. Then, they were centrifuged for 10 min at 10 000 × *g*. Supernatants were quantified by a colorimetric assay based on the Bradford method (protein assay dye reagent concentrate, Bio‐Rad, Hercules, CA, USA) using spectrophotometric absorbance measures at 595 nm (SPECTROstar Nano, BMG Labtech), with bovine serum albumin as the standard, and subsequent protein determinations. For qualitative polyacrylamide gel electrophoresis (PAGE) determination, protein extracts were prepared in buffer containing 2% SDS, 0.1% (w/v) dithiothreitol, 62.5 mmol L^−1^ Tris–HCl (pH 6.8), 12.5 mmol L^−1^ EDTA, 0.01% (w/v) bromophenol blue, and 10% (w/v) glycerol. Samples were boiled for 5 min at 95 °C and centrifuged for 5 min at 10 000 × *g*. Ten micrograms of soluble protein extracts were run on biphasic polyacrylamide gels (4% stacking gel and 10% separation gel). The gels were stained with Coomassie protein stain (InstantBlue protein stain, Expedeon, Cambridge, UK) for 2 h, rinsed with water, and photographed. For metabolic, proteomic, and C/N determination, at least six sample replicates were measured, including triplicate biological samples and triplicate technical measurements.

### Statistical analysis

The nutritional and yield data were subjected to statistical analysis utilizing the Real Statistic add‐in for Excel. Student's *t*‐test was used to compare two groups of comparison (control *vs*. treatment). Analysis of variance (two‐way ANOVA test) was used to identify significant differences in composition between different accessions and treatments. Statgraphics Centurion v18.1 software (Statgraphics Technologies, Inc., The Plains, VA, USA) was used for principal component analysis (PCA) and multivariable correlation analysis. Additionally, a factor analysis of mixed data (FAMD) was performed to explore the relationships among quantitative variables. Subsequently, a hierarchical clustering on principal components (HCPC) was applied to identify clusters within the dataset. Both analyses were conducted using the FactoMineR and factoextra packages in R (version 4.3.2). Default settings were used unless otherwise specified.^28^

### AI‐assisted technologies in the writing process

During the preparation of this work the authors used Grammarly, Inc. v2025 to improve spelling, grammar, and general editing of the manuscript. After using this tool, the authors reviewed and edited the content as needed and took full responsibility for the content of the published article.

## RESULTS

### Agro‐morphological analysis of *V. sativa* accessions

The field agro‐morphological evaluation of selected vetch accessions was based on the analysis of different quantitative traits, including measurements of grain production, and biomass and phenological data. Table 1 and Supporting Information, Table S2, show the summarized results for the evaluation of traits related to yield such as seed number, pod number, seed weight, and grain production together with phenological parameters.

**Table 1:** Mean values of the measurements of agro‐morphological traits in selected accessions of common vetch (*Vicia sativa*)

Quantitative agro‐morphological traits				
	Average	SD	Max.	Min.
Flowering				
Days to first flowering	140.7	4.1	150.3	136.0
Days to 50% flowering	142.5	5.0	152.0	136.7
Days to final flowering	163.0	4.3	173.0	158.0
Days to maturity	186.4	6.4	200.0	178.3
Pod/seed				
Number of seeds per pod	6.6	0.5	7.8	5.8
Grain weight per plant (g)	13.9	3.5	19.0	7.4
Biomass weight per plant (g)	22.2	3.6	28.4	15.4
Harvest index	0.6	0.2	0.9	0.2
100‐seed weight (g)	6.6	1.3	10.5	3.8

Differences in production and yield, both in grain and biomass, were observed among the analyzed accessions. The average number of seeds per pod was 6.6, ranging from 5.8 (SENDA) to 7.8 (BGE004375). The highest 100‐seed weight value was recorded for accession BGE005449 and the lowest for BGE016970, with 6.6 g the average value. The average value for biomass production was 22.2 g per plant, ranging from 15.4 to 28.4 g per plant. The average value for grain production was 13.9 g per plant, ranging from 7.4 to 19.0 g per plant. BGE0014946 and BGE022757 were the accessions most productive for grain and BGE000529 was the most productive for biomass. Conversely, BGE022207 and BGE025608 are the accessions less productive for biomass, and Aitana and Senda were the least productive for grain.

Regarding phenological values, relevant differences of more than 15 days were observed. Briefly, BGE000529 and SENDA could be considered late in flowering and pod maturity, Aitana was late in pod maturity, and BGE022757 early in flowering and pod maturity.

### Protein content and amino acid composition in vetch seed

**Figure 1:**
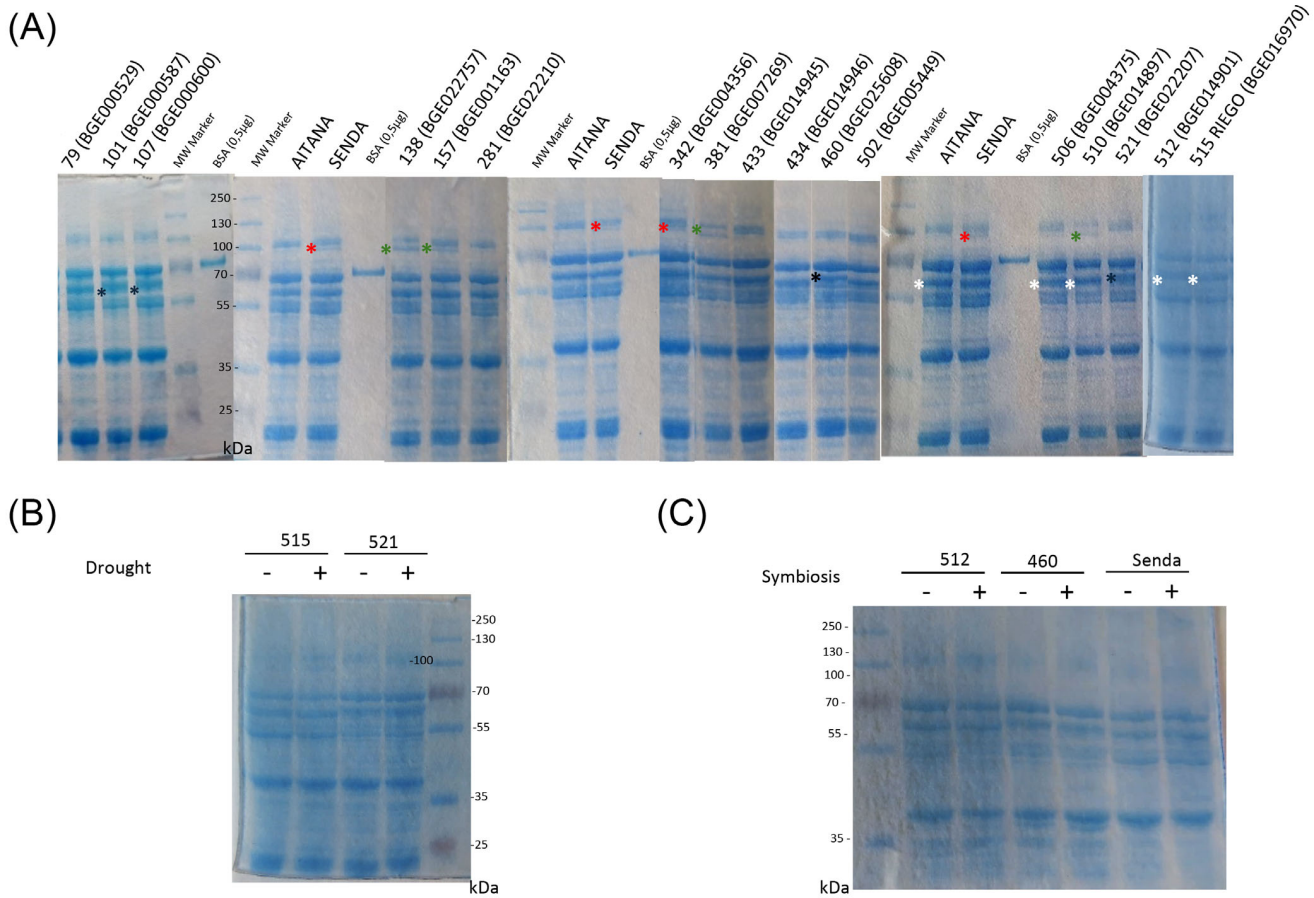
Molecular profile of protein bands analyzed in the indicated accessions by polyacrylamide gel electrophoresis, showing differences among accessions (A) or among drought (B) or symbiotic treatments (C). Asterisks in different colors indicate different extra proteins (red: 98 kDa; green: 95 kDa; black: 71 kDa; white: 68 kDa). Molecular weight standard was used for weight estimation.

The crude protein content in the seeds of common vetch is shown in Table 2. The average content of crude protein was 21.8%. The highest total protein levels were found in accession BGE000529 (30.1%), and the lowest protein value corresponded to BGE025608 (16.20%). Soluble protein content was also measured by quantitative and qualitative methods. From a qualitative analysis, no major differences were observed in soluble protein. However, specific protein bands were detected when the protein pattern was analyzed (Fig. 1(A)). Thus, we observed additional protein bands of an estimated molecular weight of 98 kDa (genotypes Senda and BGE004356), 95 kDa (genotypes BGE022757, BGE0001163, BGE0007269 and BGE0014897), 71 kDa (accessions Aitana, BGE004375, BGE014897, BGE014901 and BGE016970) and 68 kDa (accessions BGE000587, BGE000600, BGE022207 and BGE025608). No relevant differences were found in soluble protein content (Table 2; Fig. 1).

**Table 2:** Total levels of C/N, crude and soluble protein, soluble sugar and starch concentration present in the seed of the indicated accessions

Accession	CBGP code	N total (g 100 g^−1^)	C total (g 100 g^−1^)	Ratio C/N	Total crude protein (g 100 g^−1^)	Total soluble protein (mg g^−1^ DW)	Total soluble sugars (mg g^−1^ DW)	Starch (mg g^−1^ DW)
Aitana	Aitana	4.02 ± 0.36	40.65 ± 3.72	10.12	25.1 ± 2.24	3.31 ± 0.05	6.8 ± 0.3	219.9 ± 2.2
Verdor	Verdor	3.08 ± 0.27	40.57 ± 3.71	13.17	19.25 ± 1.7	4.32 ± 0.22	7.9 ± 0.4	280.5 ± 4
Senda	Senda	3.31 ± 0.29	39.72 ± 3.63	11.99	20.71 ± 1.84	4.37 ± 0.04	9.1 ± 0.4	176.8 ± 7.8
BGE000529	79	4.82 ± 0.46	41.22 ± 3.8	8.56	30.1 ± 2.89	4.25 ± 0.25	7.1 ± 0.2	244.9 ± 3.1
BGE000587	101	3.27 ± 0.32	41.21 ± 3.8	12.60	20.45 ± 2	4.64 ± 0.18	9.7 ± 0.2	265.2 ± 6.7
BGE000600	107	3.65 ± 0.32	40.7 ± 3.66	11.14	22.84 ± 2	4.58 ± 0.01	10.7 ± 0.9	263.9 ± 8.2
BGE022757	138	3.69 ± 0.32	40.91 ± 3.68	11.08	23.06 ± 2.02	4.23 ± 0.16	13.9 ± 0.6	184.6 ± 4
BGE001163	157	4.65 ± 0.41	40.28 ± 3.62	8.67	29.03 ± 2.55	3.99 ± 0.06	8.3 ± 0.7	261 ± 7.9
BGE022210	281	3.82 ± 0.33	40.49 ± 3.64	10.60	23.88 ± 2.09	4.47 ± 0.07	7.2 ± 0.8	215.5 ± 17.3
BGE004356	342	3.26 ± 0.28	40.94 ± 3.68	12.57	20.36 ± 1.74	5.04 ± 0.03	13.2 ± 0.1	189.2 ± 31.1
BGE007269	381	3.11 ± 0.27	38.8 ± 3.48	12.48	19.43 ± 1.66	4.16 ± 0.37	10.5 ± 0.8	201.9 ± 1.9
BGE014945	433	3.38 ± 0.29	40.24 ± 3.61	11.92	21.1 ± 1.81	3.93 ± 0.13	9.7 ± 0.4	243 ± 17.3
BGE014946	434	3.17 ± 0.26	39.1 ± 3.28	12.33	19.82 ± 1.6	4.77 ± 0.21	8.7 ± 0.6	164.9 ± 1.5
BGE025608	460	2.59 ± 0.21	39.3 ± 3.29	15.16	16.2 ± 1.3	4.05 ± 0.4	9.8 ± 0.7	196.7 ± 2.3
BGE005449	502	2.88 ± 0.27	39.44 ± 3.45	13.68	18.02 ± 1.69	4.23 ± 0.11	6.6 ± 0.3	296.1 ± 15.5
BGE004375	506	3.49 ± 0.32	39.79 ± 3.48	11.41	21.79 ± 2.02	4.11 ± 0.08	11 ± 1.4	266.4 ± 0.9
BGE014901	512	2.68 ± 0.25	40.45 ± 3.54	15.08	16.77 ± 1.58	4.35 ± 0.44	16.1 ± 0.8	245.1 ± 5.8
BGE016970	515	4.18 ± 0.38	41.11 ± 3.6	9.84	26.12 ± 2.4	4.31 ± 0.03	17.2 ± 0.9	188.9 ± 11.8
BGE022207	521	3.26 ± 0.29	40.96 ± 3.75	12.56	20.38 ± 1.81	4.02 ± 0.04	10.8 ± 0.2	206.4 ± 8.2
	Mean	3.49	40.31	11.84	21.81	4.32	10.48	224.1
	SE	0.60	0.75	1.82	3.74	0.31	2.94	39.5
	Maximum	4.82	41.22	15.16	30.10	5.04	17.20	296.1
	Minimum	2.59	38.80	8.56	16.20	3.93	6.60	164.9

**Figure 2:**
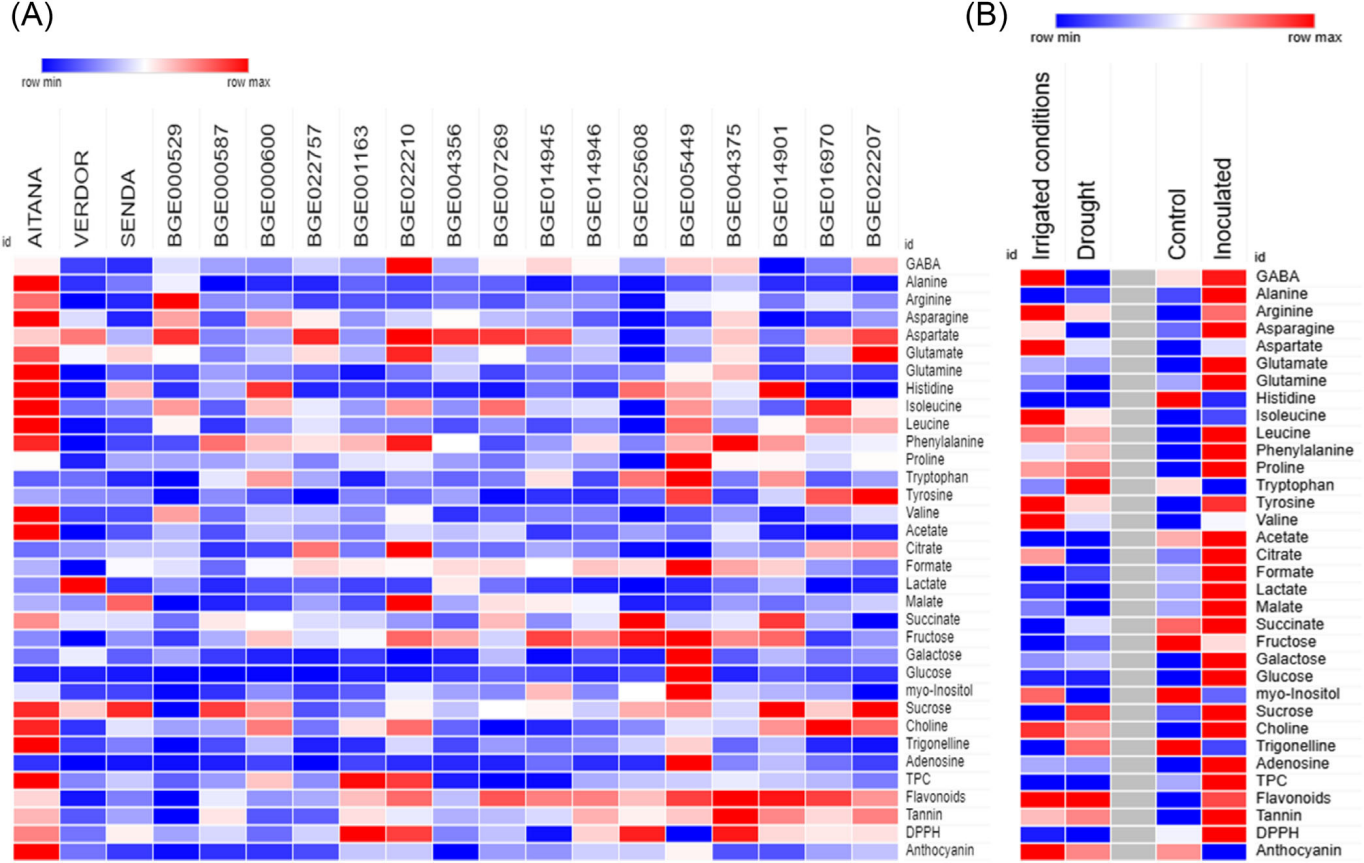
Heatmap showing relative content of amino acids and metabolites in the seeds of the different vetch accessions (A) or after different drought or rhizobia inoculation treatments (B).

Table 3 presents the detailed amino acid composition of the seed of the analyzed accessions. Aitana had the highest levels of alanine, histidine, valine, isoleucine asparagine and glutamine. BGE0022210 had the highest levels of aspartic acid and γ‐aminobutyric acid (GABA); BGE000529 had the highest levels of arginine; BGE0022207 had the highest levels of glutamic acid and tyrosine; BGE0014901 had the highest levels of histidine; BGE0004375 had the highest levels of phenylalanine; and BGE005449 had the highest levels of proline and tryptophan. Relative mean values of the different amino acids content are also indicated in the heatmap of Fig. 2(A).

**Table 3:** Amino acid concentration (μmol mg^−1^ DW) present in the seed of the indicated accessions

Accession	CBGP code	GABA	Ala	Arg	Asn	Asp	Glu	Gln	His	Ile	Leu	Phe	Pro	Trp	Tyr	Val
Aitana	Aitana	14.5 ± 0.9	44.7 ± 1.5	99.3 ± 18.2	284.7 ± 23.7	64.8 ± 2.7	153.6 ± 10.3	884.5 ± 81	42.9 ± 4.2	7.1 ± 0.1	8.1 ± 0.8	15.3 ± 1.1	63.7 ± 0.4	24 ± 1.2	8.9 ± 0.2	21.7 ± 0.5
Verdor	Verdor	8.1 ± 0.9	10.2 ± 1	24.5 ± 4.6	148.8 ± 4.8	74.9 ± 4.9	112.7 ± 10.4	399.2 ± 34.6	3.1 ± 0.1	3.7 ± 0.8	3.3 ± 0	2.8 ± 0.2	39 ± 0	27.4 ± 4.4	8.5 ± 1.8	6.8 ± 0.1
Senda	Senda	7.5 ± 1.1	15.7 ± 1.8	31.4 ± 3.7	62.7 ± 14.5	50.6 ± 3	124.6 ± 8.2	492 ± 20.2	28.6 ± 3.5	4 ± 0.3	3.9 ± 0.6	4.6 ± 1.1	53.8 ± 6.3	11.2 ± 0.4	8.5 ± 0	7 ± 0.8
BGE000529	79	13 ± 0.4	24.4 ± 6.4	120.2 ± 8	209.6 ± 30.7	82.8 ± 7.7	115.2 ± 32.1	469.7 ± 112.7	6.1 ± 1.3	5.8 ± 0	5.8 ± 0.6	4.9 ± 1.6	53.2 ± 4.7	3.4 ± 0.8	6.7 ± 0.3	16.3 ± 3.4
BGE000587	101	11.1 ± 3.3	6.7 ± 0.9	50.9 ± 9.9	83.6 ± 15.2	44.5 ± 11.1	84.6 ± 8.3	549.4 ± 40.3	16.5 ± 0.5	3.5 ± 1.4	3.7 ± 1.1	13.4 ± 1.4	53.6 ± 11.2	52.6 ± 8.6	8.5 ± 0.6	8.1 ± 1.7
BGE000600	107	10.6 ± 0.6	9.5 ± 0.1	51.9 ± 11.1	208.4 ± 8.4	48.6 ± 6.1	101.1 ± 0.6	521.2 ± 10.4	39.2 ± 0.1	5.4 ± 0	4.7 ± 0.2	11.3 ± 0.4	57.3 ± 0.2	78.3 ± 6.1	7.7 ± 0.4	11.3 ± 0.5
BGE022757	138	12.5 ± 0.7	9.3 ± 0.2	36.9 ± 1	172.6 ± 5.6	84 ± 5	122 ± 2.8	486.1 ± 4.7	5.1 ± 0.1	4.7 ± 0.1	5.4 ± 0.5	10.3 ± 0.5	50 ± 11.6	39.5 ± 0.7	6.5 ± 0.9	11.1 ± 1.2
BGE001163	157	11 ± 0.9	14.1 ± 0.4	40.2 ± 3.9	116.2 ± 0.3	46.6 ± 4.3	97.2 ± 10.3	414.6 ± 7.3	7.7 ± 0.3	4 ± 0.4	4.6 ± 0.5	11.4 ± 0	60.2 ± 2.1	9.8 ± 0.7	8.3 ± 0.1	9.2 ± 0.5
BGE022210	281	21.8 ± 2.5	12.1 ± 0.2	40 ± 10.6	144.3 ± 16.7	88.6 ± 14.3	164.3 ± 13.7	512.4 ± 19.7	6.5 ± 3.5	5.7 ± 0.3	4.5 ± 0	15.7 ± 0.1	61.6 ± 14.7	36 ± 1.6	8 ± 0.1	13.3 ± 0.1
BGE004356	342	11.4 ± 0.1	14.6 ± 1.2	48.4 ± 5.1	167.2 ± 4.9	82.9 ± 5.9	101.7 ± 2.9	590.8 ± 47.5	5.4 ± 0.3	3.9 ± 1.2	4 ± 1.1	9.6 ± 2	52.9 ± 8.6	25.1 ± 3.3	8.7 ± 1.4	6.1 ± 1
BGE007269	381	14.3 ± 0.1	13.9 ± 0.3	39.8 ± 4.5	136.2 ± 4.6	82.4 ± 3.6	115.7 ± 2.5	465.2 ± 1	3.9 ± 0.1	6.1 ± 0.1	4.6 ± 0.2	4.7 ± 0.4	48.3 ± 4.8	30.2 ± 1.1	6.7 ± 0.5	8 ± 0.1
BGE014945	433	15.3 ± 0.4	7.9 ± 0.2	52.5 ± 1	126.8 ± 5.3	79.8 ± 7.5	91.4 ± 0.3	521.6 ± 12.9	12 ± 1.5	4.5 ± 0.2	4 ± 0	7 ± 0.2	56.7 ± 5.4	64.3 ± 0.1	7.2 ± 0.2	8.9 ± 0.2
BGE014946	434	14.1 ± 1.9	13.5 ± 0.1	51.8 ± 6.8	89.6 ± 4	51.3 ± 8.5	99.5 ± 0.8	510.4 ± 6.6	7 ± 0.3	4.6 ± 1.1	4.5 ± 0.7	10.3 ± 0.5	52 ± 2.8	18.8 ± 0.7	7.1 ± 0.5	8.8 ± 1.1
BGE025608	460	11.4 ± 1.2	7.4 ± 0.5	26.1 ± 1.3	47.6 ± 5.7	29.1 ± 0.4	55.8 ± 1.1	527.8 ± 16.3	34.4 ± 0.5	2.7 ± 0.5	3.2 ± 0.2	6.2 ± 0.1	35.2 ± 8.3	87.4 ± 4.4	8 ± 0.3	4.4 ± 0.5
BGE005449	502	15.5 ± 2.9	13.4 ± 1.7	69.1 ± 1.2	83.8 ± 3.4	51.4 ± 1.1	88 ± 8.9	648.7 ± 39.3	29.6 ± 1.1	5.8 ± 1	7.1 ± 1.3	11.8 ± 0.6	91.7 ± 10	112.1 ± 12.8	12.6 ± 2	7.5 ± 0.7
BGE004375	506	15.6 ± 1.7	21 ± 0.1	71.6 ± 10.8	185.1 ± 2.4	65.9 ± 4.9	120.8 ± 0.2	707.3 ± 23.4	20.7 ± 1.2	4.4 ± 0.7	4.8 ± 0.5	16.3 ± 2	64.3 ± 0.6	29.4 ± 4.1	7.4 ± 0.3	9.7 ± 0.5
BGE014901	512	6.1 ± 1.8	9.8 ± 0.9	46.9 ± 27.7	46.2 ± 26.9	42.3 ± 18.4	71.3 ± 20.6	447.8 ± 40.4	43.1 ± 4.3	3.5 ± 1.9	5.7 ± 2.5	12.3 ± 0.5	64.5 ± 9.5	80.4 ± 2	9.4 ± 0.2	5.1 ± 1.7
BGE016970	515	9.9 ± 1.1	10.7 ± 0.7	66.4 ± 20.9	70.7 ± 3.5	67.2 ± 1.4	104.3 ± 7.9	481.4 ± 34.2	2.9 ± 0.4	6.8 ± 1	6.7 ± 0.4	8.7 ± 0.8	59.5 ± 11.5	23 ± 2.5	12.3 ± 1.1	10 ± 0.1
BGE022207	521	16.1 ± 1.1	7.8 ± 0.1	50.6 ± 8	117.9 ± 12.4	80.7 ± 3	173.8 ± 1.7	454.3 ± 3.1	2.4 ± 0.2	5.1 ± 0.6	6.5 ± 0.5	9.2 ± 1.3	63.9 ± 1	37.5 ± 0.7	13.5 ± 0.5	12 ± 0.4
	Mean	12.62	14.04	53.61	131.68	64.13	110.40	530.76	16.69	4.81	5.01	9.78	56.92	41.60	8.66	9.75
	SE	3.64	8.71	23.89	62.94	17.99	29.65	113.67	14.81	1.19	1.34	3.97	11.66	29.75	2.02	4.08
	Maximum	21.80	44.70	120.20	284.70	88.60	173.80	884.50	43.10	7.10	8.10	16.30	91.70	112.10	13.50	21.70
	Minimum	6.10	6.70	24.50	46.20	29.10	55.80	399.20	2.40	2.70	3.20	2.80	35.20	3.40	6.50	4.40

Measurements were made by analysis of ^1^H‐NMR vetch seed spectra observed in each sample.

### Sugar content and tricarboxylic acid cycle‐related metabolites in vetch seeds

Total sugar content was quantified, being BGE016970 the genotype with the highest content and Aitana the lowest. Starch was determined by enzymatic analysis, BGE005449 being the genotype with the highest content and BGE014946 with the lowest (Table 2; Fig. 2). The levels of different sugars were measured, including fructose, galactose, glucose, *myo*‐inositol and sucrose, with mean values of 1164.0, 30.4, 52.0, 100.5 and 833.8 mmol g^−1^ dry weight (DW), respectively. The accession BGE005449 had the highest levels of fructose, galactose, glucose and *myo*‐inositol, and BGE014901 had the highest level of sucrose (Table 4; Fig. 2).

**Table 4:** Sugar concentration (mmol mg^−1^ DW) present in the seed of the indicated accessions

Accession	CBGP	Fructose	Galactose	Glucose	*myo*‐Inositol	Sucrose
Aitana	Aitana	963.9 ± 103.1	31.1 ± 4.4	34.5 ± 0.4	111 ± 31.3	978 ± 96
Verdor	Verdor	744.8 ± 128.9	49.5 ± 15.5	35.1 ± 5	85.4 ± 10.5	842.8 ± 93.4
Senda	Senda	976.4 ± 52	27.3 ± 0.8	30.8 ± 4.2	86.3 ± 3.2	984.4 ± 93.9
BGE000529	79	841 ± 12.9	38.4 ± 0	20.9 ± 0	77 ± 11.3	573.3 ± 223.1
BGE000587	101	1023.7 ± 30.5	21.7 ± 5.3	19 ± 1.6	83.6 ± 23.6	962.5 ± 64.4
BGE000600	107	1240.4 ± 133.5	17.9 ± 4.6	21.6 ± 4.1	97.8 ± 9.8	885 ± 148.7
BGE022757	138	1083.2 ± 97.6	15.7 ± 2.1	19.1 ± 1.4	86.6 ± 3.8	646 ± 24.2
BGE001163	157	1138.5 ± 128	22.1 ± 0.3	33.7 ± 4.2	90.1 ± 4.8	686.8 ± 18.9
BGE022210	281	1387.4 ± 89.8	12.5 ± 0.1	29.3 ± 11.6	112.5 ± 31.5	801.9 ± 28.6
BGE004356	342	1286 ± 121.4	17.8 ± 2.6	50.1 ± 1.1	100.6 ± 13.7	745.7 ± 45.5
BGE007269	381	1074.7 ± 23.9	41.9 ± 1.1	67.7 ± 1.6	97.3 ± 5	791.4 ± 12.1
BGE014945	433	1439.6 ± 48.7	13.1 ± 1.4	59 ± 8	125.9 ± 10.2	808.2 ± 14.3
BGE014946	434	1332.4 ± 9.7	24.5 ± 6.1	39.4 ± 4	97.3 ± 0	755.5 ± 32.5
BGE025608	460	1505.1 ± 21.2	17.4 ± 0.4	57.2 ± 17.8	114.9 ± 7.1	864.9 ± 29.1
BGE005449	502	1540.2 ± 145.4	92.6 ± 38.4	274.8 ± 66.4	153.9 ± 9.7	887.5 ± 46
BGE004375	506	1330.3 ± 44.4	26.5 ± 7.3	36.6 ± 14.9	107.9 ± 5.6	759.8 ± 24.3
BGE014901	512	1381.3 ± 307.4	41.7 ± 8.7	64.9 ± 51.4	103.7 ± 16.3	1019 ± 109.6
BGE016970	515	840 ± 63.3	30.8 ± 2.2	49 ± 2.2	101.3 ± 11.7	835.7 ± 75.9
BGE022207	521	986.6 ± 8.7	34.8 ± 0	44.4 ± 0.3	76.4 ± 3.3	1014 ± 20.6
	Mean	1163.97	30.38	51.95	100.50	833.81
	SE	239.71	18.41	56.05	18.56	124.93
	Maximum	1540.20	92.60	274.80	153.90	1019.00
	Minimum	744.80	12.50	19.00	76.40	573.30

Measurements were made by analysis of ^1^H‐NMR vetch seed spectra observed in each sample.

The levels of metabolites of the tricarboxylic acid cycle (TCA), one of the major pathways of carbohydrate catabolism, were measured, including acetate, citrate, formate, lactate, malate and succinate, with mean values of 8.67, 199.8, 3.65, 17.31, 100.57 and 7.87 mmol g^−1^ DW, respectively. Aitana had the highest level of acetate; BGE022210 had the highest levels of citrate and malate; BGE005449 had the highest level of formate; and BGE025608 had the highest level of succinate (Table 5; Fig. 2).

**Table 5:** Total TCA organic acid content (mmol mg^−1^ DW) present in the seed of the indicated accessions

Accesssion	CBGP code	Acetate	Citrate	Formate	Lactate	Malate	Succinate	Choline	Trigonelline	Adenosine
Aitana	Aitana	19.7 ± 0.1	161.7 ± 1.3	3.2 ± 0.4	19 ± 1.8	101.4 ± 14.7	9.8 ± 0.8	235.1 ± 14.7	55.8 ± 5	3.1 ± 0.5
Verdor	Verdor	4.5 ± 1	186.3 ± 16.3	2.1 ± 0.5	39.9 ± 34.6	94.4 ± 10.8	7.8 ± 2.2	128.2 ± 7.2	20.3 ± 4.3	1.7 ± 0.3
Senda	Senda	7 ± 0	211.7 ± 15.4	3.7 ± 0.2	14.3 ± 2.2	154.1 ± 10.3	7.7 ± 0.4	172.4 ± 11.1	25.2 ± 2.1	2.2 ± 0.2
BGE000529	79	10 ± 2	210.4 ± 2.1	3.5 ± 1.3	19.5 ± 5.2	62.1 ± 15.8	6.3 ± 0.7	155.8 ± 39.7	15.2 ± 6.2	2.1 ± 0
BGE000587	101	6.8 ± 1	128.3 ± 14.5	2.8 ± 0.3	13.4 ± 2.3	69.8 ± 15.2	8.5 ± 1	157.1 ± 15.6	21.1 ± 0.8	2.5 ± 0.3
BGE000600	107	10.2 ± 2.2	139.6 ± 7.9	3.6 ± 0.3	15.9 ± 1	78.4 ± 7.6	8.2 ± 1.7	211.9 ± 6.1	30.2 ± 0	3.6 ± 1.8
BGE022757	138	9.1 ± 1.3	323.1 ± 5.9	4 ± 0.2	17.1 ± 0.7	98.3 ± 2.8	7.8 ± 0.6	143.4 ± 4.2	17.7 ± 0.6	1.7 ± 0.5
BGE001163	157	8.1 ± 3	167.7 ± 4.5	3.8 ± 0.3	14.8 ± 0.3	78.9 ± 7.2	7.6 ± 0.2	187.1 ± 3.8	18.7 ± 1.1	3.6 ± 0.5
BGE022210	281	11 ± 0.7	390.7 ± 1.5	3.7 ± 0.6	14.9 ± 1.8	175.9 ± 18.1	6.8 ± 0.6	216.2 ± 12	32.2 ± 0.1	2.9 ± 0.3
BGE004356	342	10.2 ± 2.7	173.5 ± 8.1	3.9 ± 0.2	26.8 ± 3.2	99.9 ± 10.3	5.9 ± 0.4	139.7 ± 6	20.9 ± 0.9	2.8 ± 0.2
BGE007269	381	10.9 ± 0.1	161.4 ± 3.6	3.9 ± 0.6	17.7 ± 2.9	126.3 ± 7.5	9.1 ± 1.2	114.1 ± 0.5	27.5 ± 0.2	2.6 ± 0.1
BGE014945	433	6 ± 0.2	196.3 ± 2.1	3.7 ± 0.4	14.3 ± 0.6	122.8 ± 10.1	6.6 ± 0	124.4 ± 2.1	25 ± 0.3	4.7 ± 0.6
BGE014946	434	7.6 ± 1.2	175.6 ± 0.5	4.1 ± 0.7	12.9 ± 2.5	116.3 ± 0.1	7.8 ± 1.4	140.7 ± 3.4	26.2 ± 0.2	2.9 ± 0.7
BGE025608	460	9.2 ± 1.4	106.5 ± 3.4	3.9 ± 0	11.3 ± 1.5	68.5 ± 4.3	11.8 ± 0.7	149.6 ± 2.5	31.8 ± 0.3	2.9 ± 0.3
BGE005449	502	7.8 ± 0.1	99.9 ± 8.3	5.3 ± 0.9	13.6 ± 1.2	72.5 ± 5.1	7.4 ± 2.3	172.3 ± 12.7	39.3 ± 2.9	15.6 ± 2.1
BGE004375	506	11.3 ± 1.5	197.6 ± 4.5	4.3 ± 0.9	17.4 ± 1.3	97.8 ± 6.1	7.8 ± 0.2	167.4 ± 4.3	23.4 ± 1.6	5.3 ± 1.2
BGE014901	512	5.4 ± 2	178.4 ± 12	4 ± 0.1	21.5 ± 1.6	87 ± 18.1	11 ± 2	206.9 ± 19	30 ± 3.5	6.9 ± 3.3
BGE016970	515	4.5 ± 0.1	290.1 ± 36.4	3.1 ± 0.8	11.2 ± 0.4	97.9 ± 7.3	7.1 ± 0.9	244.8 ± 19.7	18 ± 1.7	4.8 ± 1.1
BGE022207	521	5.5 ± 0.3	299.1 ± 4.8	2.8 ± 0.1	13.3 ± 0.7	108.5 ± 0.9	4.6 ± 0.2	217.2 ± 4	16.3 ± 0.6	3.5 ± 0.3
	Mean	8.67	199.89	3.65	17.31	100.57	7.87	172.858	26.042	3.968
	SE	3.47	75.73	0.67	6.65	29.30	1.70	39.220	9.605	3.108
	Maximum	19.70	390.70	5.30	39.90	175.90	11.80	244.800	55.800	15.600
	Minimum	4.50	99.90	2.10	11.20	62.10	4.60	114.100	15.200	1.700

Measurements were made by analysis of ^1^H‐NMR vetch seed spectra observed in each sample.

### Antioxidants and ANFs in vetch seeds

Analyses of total phenolic components indicated a mean value of 2.88 mg GAE g^−1^ DW. Commercial variety AITANA and local variety BGE001163 had the highest TPC, and BGE007269 and BGE014945 the lowest. Flavonoid measurement showed a mean value of 8.64 mg RE g^−1^ DW. Local varieties BGE004375 and BGE014901 had the highest flavonoid content, and BGE000529 and Verdor the lowest. Total tannin content was also determined with a mean value of 234.22 mg CE g^−1^ DW. Local variety BGE004375 had the highest tannin content and BGE000529 the lowest. The mean of anthocyanin content was measured, indicating a mean value of 0.89 mg g^−1^ DW, with Aitana and BGE004356 being the varieties with highest and the lowest values, respectively. Regarding antioxidant activity DPPH, measurements showed a mean of 7.19 μg TE g^−1^ DW. BGE001163 had the highest DPPH content, and BGE005449 and BGE0014945 the lowest (Table 6; Fig. 2A). Levels of trigonelline, an alkaloid with a relevant role in legumes, were also measured, with a mean of 26.04 mmol g^−1^ DW, Aitana being the accession with highest levels (Table 5; Fig. 2A).

**Table 6:** Total phenolic content, total flavonoid content, total tannin contents, anthocyanins and DPPH activity in different selected accessions

Accession	CBGP code	Total phenolic compounds	Flavonoids	Total tannin content	DPPH	Anthocyanins
		(mg GAE g^−1^ DW)	mg RE g^−1^ DW	mg C E g^−1^ DW	μg TE g^−1^ DW	mg g^−1^ DW
Aitana	Aitana	5.13 ± 0.14	8.65 ± 0.27	269.32 ± 21.98	8.39 ± 0.83	2.37 ± 0.44
Verdor	Verdor	2.47 ± 0.28	7.5 ± 0.4	169.95 ± 11.4	5.87 ± 1.23	0.76 ± 0.02
Senda	Senda	2.95 ± 0.9	7.92 ± 0.19	200.93 ± 2.25	7.31 ± 0.57	0.52 ± 0.06
BGE000529	79	2.22 ± 0.16	7.39 ± 0.24	132.91 ± 10.58	6.27 ± 0.49	0.33 ± 0.09
BGE000587	101	2.62 ± 0.42	8.41 ± 0.08	250.96 ± 3.85	6.8 ± 0.53	0.49 ± 0.04
BGE000600	107	3.73 ± 1.22	8.04 ± 0.15	168.02 ± 2.28	5.77 ± 0.74	0.5 ± 0.03
BGE022757	138	2.55 ± 0.86	8.14 ± 0.18	172.62 ± 6.84	6.76 ± 0.68	0.59 ± 0.06
BGE001163	157	5.07 ± 1.61	8.7 ± 0.13	251.49 ± 15.37	9.62 ± 0.91	1.1 ± 0.09
BGE022210	281	4.72 ± 1.7	9.09 ± 0.33	230.96 ± 8.21	9 ± 0.58	1.11 ± 0.09
BGE004356	342	1.75 ± 0.56	8.22 ± 0.19	204.83 ± 15.75	6.01 ± 0.42	0.29 ± 0.01
BGE007269	381	1.53 ± 0.87	9.14 ± 0.49	211.28 ± 34.46	6.58 ± 0.28	0.93 ± 0.12
BGE014945	433	1.59 ± 0.78	8.96 ± 0.33	223.93 ± 2.79	4.9 ± 0.2	0.89 ± 0.07
BGE014946	434	2.76 ± 0.97	8.99 ± 0.24	272.28 ± 14.9	7.65 ± 0.89	1.13 ± 0.07
BGE025608	460	2.93 ± 0.59	8.69 ± 0.22	244.87 ± 16.32	9.3 ± 1.01	1.13 ± 0.06
BGE005449	502	2.99 ± 0.69	9.24 ± 0.2	262.32 ± 14.98	4.73 ± 0.25	1.37 ± 0.06
BGE004375	506	3.19 ± 0.65	9.49 ± 0.32	346.94 ± 12.69	9.39 ± 1.39	0.63 ± 0.05
BGE014901	512	2.95 ± 0.22	9.45 ± 0.16	288.86 ± 32.49	7.5 ± 0.33	0.62 ± 0.02
BGE016970	515	2.83 ± 0.44	9.25 ± 0.21	255.77 ± 21.39	7.41 ± 0.89	0.95 ± 0.04
BGE022207	521	2.41 ± 0.5	8.88 ± 0.14	292.01 ± 25.45	7.43 ± 0.34	1.08 ± 0.04
	Mean	2.97	8.64	234.22	7.19	0.89
	SE	1.05	0.63	52.09	1.46	0.47
	Maximum	5.13	9.49	346.94	9.62	2.37
	Minimum	1.53	7.39	132.91	4.73	0.29

Values are expressed per gram of seed dry weight (DW).

Abbreviations: CE, catechin equivalent; GAE, gallic acid equivalent; RE, rutin equivalent; TE, Trolox equivalent.

### Mineral composition in vetch seed

**Figure 3:**
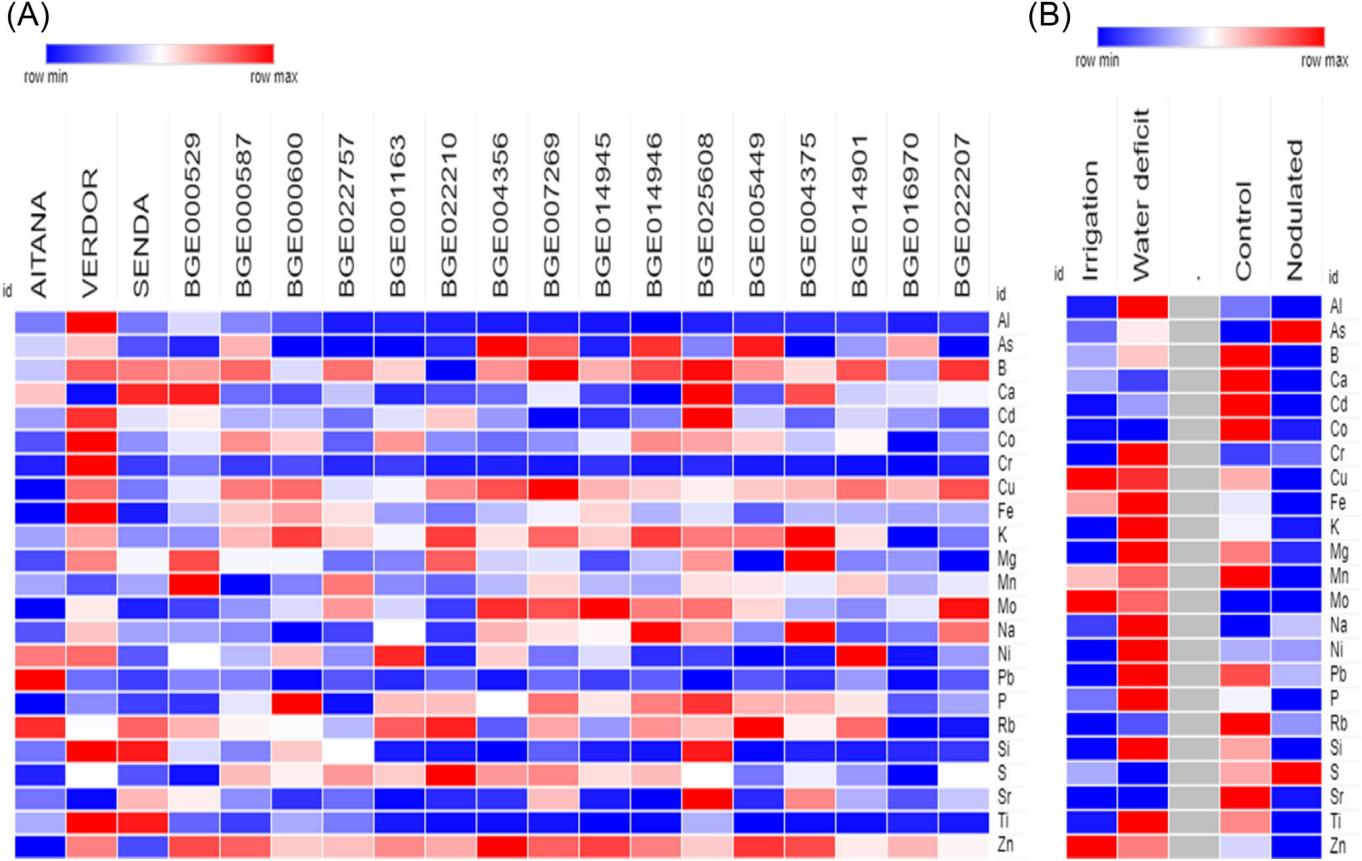
Heatmap showing relative content of indicated elements in the seeds of the different vetch accessions (A) or after different drought or rhizobia inoculation treatments (B).

Table 7 shows the levels of 23 minerals (in addition to Be, Bi, La, Li, Sb, Se, Ti and V, with values below the detection level) in the 19 analyzed accessions. Statistical analysis of the results indicates that the levels of macroelements (K, Mg, Ca, P and S), as well as also some microelements (B, Mn, Zn) among the different genotypes remained quite uniform and the content of these minerals showed low variability (<12% of variation) with a narrow range of values. Other minerals, such as Cd, Co, Cu, Fe, Mo, Na, Ni, Rb and Sb, presented moderate variability (between 12% and 40%). Finally, other minor minerals, such as Al, As, Cr, Pb, Si and Ti, had a wide range variation (>40%). It is noteworthy that Aitana variety presented the lowest levels for various elements such as Cu, Fe, Mo, Zn and P; and BGE016970 the lowest content of Co, Cr, K, Rb and S elements. Verdor variety stood out as the accession with the highest content of Al, Co, Cr, Fe, Si and Ti (Table 7; Fig. 3).

**Table 7:** Total mineral composition in different selected accessions on dried seeds

Accession	Al	As	B	Ca	Cd	Co	Cr	Cu	Fe	K	Mg	Mn
	(mg kg^−1^)	(mg kg^−1^)	(mg kg^−1^)	(g kg^−1^)	(mg kg^−1^)	(mg kg^−1^)	(mg kg^−1^)	(mg kg^−1^)	(mg kg^−1^)	(g kg^−1^)	(g kg^−1^)	(mg kg^−1^)
Aitana	8.63 ± 0.85	0.1 ± 0.03	4.73 ± 0.55	1.2 ± 0.2	0.017 ± 0.001	0.16 ± 0.01	0.37 ± 0.06	6.1 ± 0.8	55.5 ± 5	11.4 ± 1.2	1.2 ± 0.2	35.89 ± 4.08
Verdor	29.79 ± 2.79	0.15 ± 0.01	5.91 ± 0.7	0.9 ± 0.2	0.032 ± 0.002	0.51 ± 0.04	4.27 ± 0.49	12.2 ± 1.6	146.7 ± 13.3	12.4 ± 1.6	1.4 ± 0.2	32.38 ± 3.8
Senda	8.48 ± 0.83	0.04 ± 0.01	5.7 ± 0.66	1.3 ± 0.3	0.02 ± 0.002	0.21 ± 0.02	0.6 ± 0.07	8 ± 1	59.7 ± 5.4	11.2 ± 1.7	1.3 ± 0.2	35.74 ± 4.8
BGE000529	13.78 ± 1.32	0.02 ± 0	5.53 ± 0.63	1.3 ± 0.3	0.02 ± 0.002	0.28 ± 0.02	1.09 ± 0.14	9.6 ± 1.2	89.6 ± 8.2	11.2 ± 0.9	1.4 ± 0.3	49.7 ± 5.57
BGE000587	9.17 ± 0.33	0.16 ± 0	5.83 ± 0.72	0.9 ± 0.3	0.023 ± 0.002	0.39 ± 0.03	0.58 ± 0.08	12 ± 1.5	110.8 ± 10.1	12.3 ± 1.3	1.3 ± 0.2	29 ± 4.97
BGE000600	6.8 ± 0.22	<0.01	4.84 ± 0.62	0.9 ± 0.1	0.018 ± 0.001	0.35 ± 0.02	0.75 ± 0.1	12.2 ± 1.6	118.6 ± 10.8	13 ± 1.5	1.3 ± 0.3	34.08 ± 4.37
BGE022757	3.27 ± 0.37	<0.01	5.79 ± 0.7	1 ± 0.3	0.018 ± 0.002	0.17 ± 0.01	0.44 ± 0.07	9.4 ± 1.2	106 ± 9.7	12.2 ± 1.9	1.2 ± 0.3	44.63 ± 4.93
BGE001163	3.78 ± 0.46	<0.01	5.29 ± 0.63	0.9 ± 0.3	0.015 ± 0.001	0.39 ± 0.03	0.62 ± 0.08	9.8 ± 1.3	83.3 ± 7.6	11.8 ± 1.1	1.2 ± 0.3	34.74 ± 4.5
BGE022210	3.63 ± 0.45	0.02 ± 0.01	3.67 ± 0.43	0.9 ± 0.2	0.02 ± 0.002	0.21 ± 0.01	0.34 ± 0.04	11.8 ± 1.5	76.5 ± 7	13 ± 1.2	1.4 ± 0.2	33.23 ± 4.59
BGE004356	3.27 ± 0.52	0.25 ± 0	5.63 ± 0.69	1 ± 0.2	0.024 ± 0.002	0.18 ± 0.01	0.38 ± 0.04	12.7 ± 1.6	89.1 ± 8.1	12.1 ± 1.7	1.3 ± 0.2	36.52 ± 5.83
BGE007269	3.21 ± 0.37	0.2 ± 0.01	6.38 ± 0.79	1.1 ± 0.1	0.017 ± 0.001	0.21 ± 0.01	0.3 ± 0.05	13.9 ± 1.8	98.1 ± 8.9	12.8 ± 1.7	1.3 ± 0.2	40.91 ± 4.87
BGE014945	3.02 ± 0.52	0.02 ± 0.01	5.46 ± 0.64	0.9 ± 0.2	0.009 ± 0.001	0.29 ± 0.02	0.53 ± 0.06	11.2 ± 1.5	108.4 ± 9.9	12.2 ± 1.2	1.2 ± 0.2	36.38 ± 4.7
BGE014946	1.85 ± 0.39	0.23 ± 0.02	6 ± 0.73	0.8 ± 0.3	0.011 ± 0.001	0.4 ± 0.03	0.36 ± 0.05	10.7 ± 1.4	87.1 ± 7.9	13.1 ± 2.2	1.3 ± 0.2	35.88 ± 5.93
BGE025608	3.51 ± 0.31	0.07 ± 0	6.3 ± 0.74	1.4 ± 0.3	0.015 ± 0.002	0.38 ± 0.03	0.45 ± 0.07	10.3 ± 1.3	95.6 ± 8.7	12.6 ± 1.6	1.4 ± 0.3	40.65 ± 5.47
BGE005449	4.39 ± 0.53	0.24 ± 0.01	5.61 ± 0.68	0.9 ± 0.2	0.034 ± 0.002	0.35 ± 0.02	0.33 ± 0.05	10.9 ± 1.4	71.9 ± 6.6	12.6 ± 1.1	1.1 ± 0.1	40.23 ± 6.43
BGE004375	4.36 ± 1.53	<0.01	5.22 ± 0.63	1.3 ± 0.2	0.019 ± 0.001	0.26 ± 0.02	0.32 ± 0.05	11 ± 1.4	88.1 ± 8	13.4 ± 1.7	1.5 ± 0.2	38.49 ± 5.03
BGE014901	5.04 ± 0.72	0.08 ± 0.01	5.94 ± 0.74	1 ± 0.3	0.014 ± 0.001	0.31 ± 0.02	0.23 ± 0.04	12.1 ± 1.6	86.9 ± 7.9	12 ± 1	1.2 ± 0.2	41.49 ± 6.04
BGE016970	3.41 ± 0.9	0.17 ± 0	4.54 ± 0.55	1.1 ± 0.2	0.02 ± 0.002	0.1 ± 0.01	0.13 ± 0.02	11.1 ± 1.4	84.1 ± 7.7	10.4 ± 1.6	1.2 ± 0.2	35.98 ± 5.94
BGE022207	5.12 ± 0.68	<0.01	6.1 ± 0.74	1.1 ± 0.3	0.017 ± 0.001	0.22 ± 0.02	0.43 ± 0.05	12.6 ± 1.6	85.7 ± 7.8	11.1 ± 1	1.1 ± 0.1	38.32 ± 4.55
Mean	6.55	0.12	5.50	1.05	0.019	0.28	0.66	10.92	91.68	12.15	1.28	37.59
SE	6.34	0.09	0.67	0.18	0.006	0.10	0.90	1.81	20.88	0.80	0.11	4.68
Maximum	29.79	0.25	6.38	1.40	0.034	0.51	4.27	13.86	146.74	13.40	1.50	49.70
Minimum	1.85	0.02	3.67	0.80	0.009	0.10	0.13	6.09	55.45	10.40	1.10	29.00

Accession	Mo	Na	Ni	Pb	P	Rb	Si	S	Sr	Ti	Zn
	(mg kg^−1^)	(mg kg^−1^)	(mg kg^−1^)	(mg kg^−1^)	(g kg^−1^)	(mg kg^−1^)	(mg kg^−1^)	(g kg^−1^)	(mg kg^−1^)	(mg kg^−1^)	(mg kg^−1^)
Aitana	16.85 ± 2.33	22.3 ± 8.6	1.21 ± 0.09	0.92 ± 0.08	3.8 ± 0.7	93.4 ± 10.5	22.8 ± 2.7	1.8 ± 0.2	4.1 ± 0.55	0.88 ± 0.11	42.6 ± 7.8
Verdor	36.72 ± 4.94	51 ± 14.8	1.24 ± 0.09	0.2 ± 0.03	4.3 ± 1.3	69.5 ± 7.8	66.3 ± 8	2.1 ± 0.3	3.39 ± 0.46	2.32 ± 0.29	65 ± 11.9
Senda	19.05 ± 3.11	32 ± 14.3	0.51 ± 0.06	0.11 ± 0.02	4 ± 0.7	87.3 ± 9.8	63.5 ± 7.4	1.9 ± 0.3	5.42 ± 0.76	2.21 ± 0.24	47 ± 8.7
BGE000529	21.41 ± 3.28	32.1 ± 11.1	0.89 ± 0.1	0.24 ± 0.07	3.9 ± 1.1	78.7 ± 8.9	33.9 ± 4	1.8 ± 0.2	5.06 ± 0.7	0.58 ± 0.06	68.7 ± 12.7
BGE000587	27.49 ± 3.4	28.8 ± 13.8	0.74 ± 0.1	0.23 ± 0.04	4.6 ± 0.6	71 ± 8	24.4 ± 3.5	2.2 ± 0.3	4.26 ± 0.61	0.42 ± 0.15	67 ± 12.3
BGE000600	32.34 ± 3.81	11.9 ± 9.4	1.05 ± 0.06	0.01 ± 0.06	5.6 ± 1.5	69.8 ± 7.8	44 ± 5	2.1 ± 0.3	3.65 ± 0.47	0.88 ± 0.15	60.9 ± 11.2
BGE022757	42.39 ± 5.03	19.9 ± 11.6	0.63 ± 0.08	0.15 ± 0.02	3.8 ± 0.5	62.5 ± 7	38.4 ± 4.6	2.2 ± 0.3	4.04 ± 0.52	0.68 ± 0.14	61.7 ± 11.4
BGE001163	32.03 ± 3.52	43.3 ± 15	1.4 ± 0.07	0.07 ± 0.02	4.9 ± 0.8	88.5 ± 10	12.8 ± 1.9	2.1 ± 0.3	3.4 ± 0.47	0.25 ± 0.11	64.6 ± 11.9
BGE022210	21.3 ± 3.1	18.2 ± 11	0.37 ± 0.1	0.19 ± 0.03	4.9 ± 0.7	94.8 ± 10.7	12.5 ± 1.5	2.4 ± 0.3	3.62 ± 0.51	0.19 ± 0.08	62.7 ± 11.5
BGE004356	50.13 ± 5.48	52.8 ± 12.3	1.01 ± 0.1	0.03 ± 0.04	4.7 ± 1	51.5 ± 5.8	9.7 ± 1.1	2.2 ± 0.3	3.65 ± 0.47	0.21 ± 0.04	73 ± 13.4
BGE007269	47.66 ± 6.54	47 ± 14.1	0.57 ± 0.1	0.19 ± 0.04	5.2 ± 0.7	79.6 ± 9	20.5 ± 2.8	2.2 ± 0.3	5.37 ± 0.71	0.23 ± 0.04	66.9 ± 12.3
BGE014945	53.03 ± 5.99	44.4 ± 13.7	0.81 ± 0.06	0.11 ± 0.06	4.8 ± 1.2	58.5 ± 6.6	13.1 ± 1.9	2.1 ± 0.3	3.5 ± 0.48	0.16 ± 0.11	69 ± 12.7
BGE014946	44.34 ± 5.39	74.5 ± 18.6	0.4 ± 0.07	0.17 ± 0.04	5.2 ± 1.4	82.3 ± 9.3	11.8 ± 1.7	2.2 ± 0.3	3.32 ± 0.48	0.17 ± 0.1	65.3 ± 12
BGE025608	45.06 ± 5.21	55.2 ± 12.9	0.45 ± 0.06	<0.01	5.4 ± 0.9	77 ± 8.7	63.9 ± 7.8	2.1 ± 0.2	6.54 ± 0.87	0.91 ± 0.13	61 ± 11.2
BGE005449	37.88 ± 4.15	29.4 ± 9.3	0.3 ± 0.1	0.16 ± 0.06	5 ± 0.7	98.6 ± 11.1	10.3 ± 1.3	1.9 ± 0.3	3.58 ± 0.52	0.15 ± 0.11	69.9 ± 12.9
BGE004375	29.56 ± 4.57	75.4 ± 16.5	0.35 ± 0.11	0.09 ± 0.06	5 ± 1	71.9 ± 8.1	13.5 ± 1.9	2 ± 0.3	5.7 ± 0.75	0.2 ± 0.03	68.5 ± 12.6
BGE014901	26.86 ± 4.28	22.9 ± 8.5	1.5 ± 0.06	0.28 ± 0.07	4.8 ± 0.9	86.8 ± 9.8	13 ± 1.6	1.9 ± 0.3	4.44 ± 0.6	0.2 ± 0.12	59 ± 10.8
BGE016970	33.18 ± 5.57	27.2 ± 13.2	0.35 ± 0.08	0.01 ± 0.05	4.1 ± 0.6	41.4 ± 4.7	14.4 ± 2	1.8 ± 0.3	3.87 ± 0.51	0.28 ± 0.08	62.5 ± 11.5
BGE022207	52.12 ± 5.68	61.2 ± 13.1	0.66 ± 0.1	0.16 ± 0.06	4.4 ± 1.1	44 ± 5	16 ± 2.3	2.1 ± 0.2	4.59 ± 0.62	0.25 ± 0.08	58.7 ± 10.8
Mean	35.23	39.45	0.76	0.18	4.65	74.07	26.55	2.06	4.29	0.59	62.84
SE	11.52	18.67	0.38	0.20	0.55	16.64	19.48	0.17	0.93	0.65	7.48
Maximum	53.03	75.42	1.50	0.92	5.60	98.64	66.25	2.40	6.54	2.32	73.00
Minimum	16.85	11.94	0.30	0.01	3.80	41.43	9.74	1.80	3.32	0.15	42.59

Measures were made by analysis of inductively coupled plasma optical emission spectrometry (ICS‐OES) vetch seed spectra observed in each sample. The content of Be, Bi, La, Li, Sb, Se, Tl and V was also measured with values below detection level (<0.01 mg kg^−1^).

### Multivariate analysis

**Figure 4:**
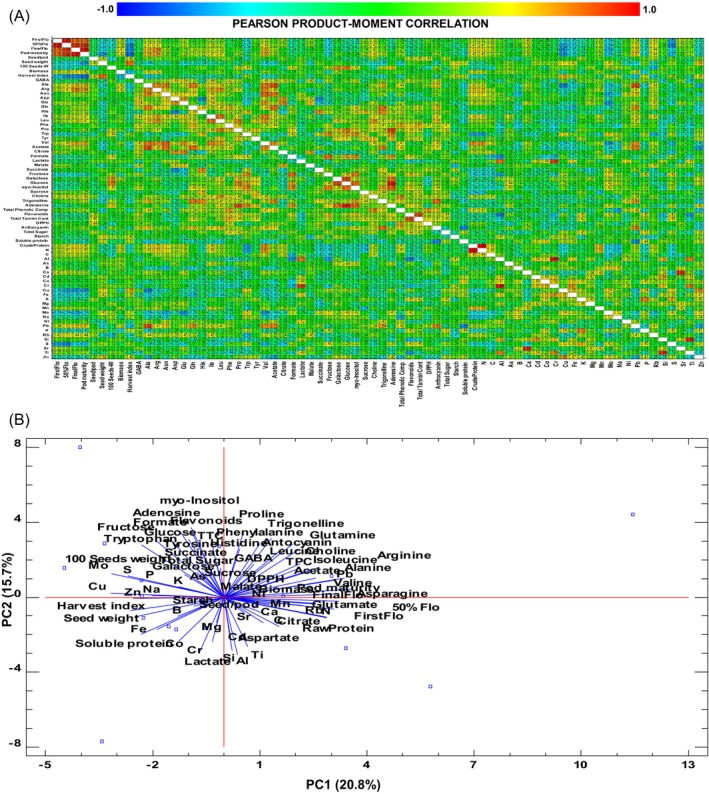
Multi‐variable analysis: (A) Pearson product–moment correlations among the different analyzed parameters; (B) factor analysis of mixed data biplot of indicated parameters.

**Figure 5:**
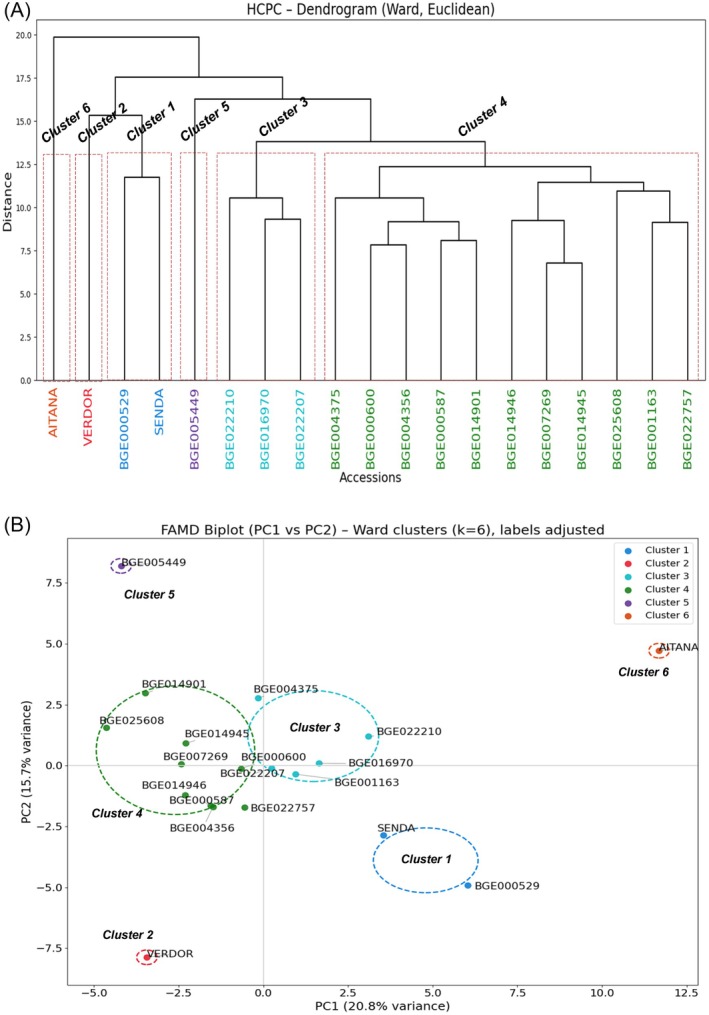
Factor analysis of mixed data (FAMD) and hierarchical clustering of principal components (HCPC): (A) dendrogram based on Ward (square Euclidean distance) of the studied accessions (mean values); the numbers on the vertical axis refer to the distance level calculated based on differences among the 19 accession contents of 72 selected components; (B) FAMD biplot.

In this study more than 80 parameters were analyzed, including quantification of 30 minerals, 15 amino acids, and various sugars and primary and secondary metabolites, with biochemical determination of several antioxidants, protein and carbohydrate composition, in addition to nine agro‐morphological traits. These measurements were performed over 19 accessions. Due to the size and complexity of the obtained data, multi‐variable analysis methods were applied to examine correlations among the different analyzed parameters (Fig. 4). Correlations were found among the different parameters related to phenology and production. Some amino acid levels also correlated among them (Ala, Arg, Asn, Gln and Val, or Leu and Ile). Levels of glucose and galactose, fructose and *myo*‐inositol and adenine also showed high levels of correlation. Also, there was a high correlation between tannins and flavonoids, and between trigonelline and anthocyanins. Pb levels and some amino acids were also correlated. Finally, some pairs of elements were also correlated, such as Ti–Sb, Zn–Cu, Sr–Ca, P–K and Al–Cr. Interestingly, a negative association was observed between the parameters of soluble protein and anthocyanin, Cu and Gln, and biomass and Mn (Fig. 4(A),(B)). In order to find diversity patterns among accessions, the data were analyzed using PCA, FAMD and HCPC. The analyses indicate that, from the 72 original variables, the 16 extracted components collectively accounted for 97.65% of the total variability in the dataset. Among these, principal component 1 (PC1) and principal component 2 (PC2) together explained more than 36.3% of the total variance, with PC1 contributing 20.8% and PC2 contributing 15.7% (Supporting Information, Table S3). The clustering analysis of these parameters allows the construction of a dendrogram of distance among accessions based on the ‘nearest‐neighbor method’. Cluster analysis revealed that the accessions were divided into six (*k* = 6) main subclusters (Fig. 5). The largest group, cluster 4, included 11 accessions (BGE004375, BGE000600, BGE004356, BGE000587, BGE014901, BGE014946, BGE007269, BGE014945, BGE025608, BGE001163 and BGE022757). Cluster 3 comprised three accessions (BGE016970, BGE022207 and BGE022210), while cluster 1 contained two accessions (BGE000529 and Senda). Clusters 2 (Verdor), 5 (BGE005449) and 6 (Aitana) each included a single accession. Accessions in sub‐cluster 1 showed late flowering, high biomass, high amino acids, moderate phenolics and intermediate antioxidant capacity. Cluster 2 (Verdor) displayed the earliest flowering, the highest seed‐to‐pod ratio, high starch, the lowest phenolic compounds and high soluble protein. Sub‐cluster 3 was similar to cluster 5 but presented higher starch and tannins, moderate antioxidant activity and higher mineral content. Sub‐cluster 4 was characterized by early flowering, moderate biomass, high seed weight, intermediate phenolic compounds and moderate antioxidant activity. Sub‐cluster 5 (BGE005449) showed intermediate flowering, high sucrose and sugars, moderate phenolics and balanced amino acids. Finally, sub‐cluster 6 (Aitana) presented very late flowering, extremely high phenolic compounds, the highest antioxidant activity, and high flavonoids and tannins.

### Impact of water deprivation on nutritional composition

In addition to variations among different accessions, we explored the effect of different treatments, such as drought or symbiosis, on nutritional composition. Our data indicate that drought promotes a decrease of total N content and total proteins, both soluble and total crude proteins, with a significant increase in some essential amino acids such as Phe and Trp but a decrease in Asn and GABA levels (Tables 8(A) and 9(A)). Regarding carbohydrate levels, a general rise in soluble sugars and starch was observed after drought treatments (Table 8(A)), with a specific sugar increase of 15% in sucrose levels (Table 10(A)). Curiously, the water deficit also promoted a decrease in citrate levels and an increase of more than 33% in trigonelline levels (Table 11(A)). Analyzing the ANF composition after drought treatments did not show significant differences except for anthocyanin levels (Table 12(A)). Mineral composition after drought stress was also analyzed, and a significant increase in the levels of the macroelements P and K, as well as in the microelements Fe and Mo, was observed (Table 13(A)).

**Table 8:** Total levels of C/N, crude and soluble protein, soluble sugar and starch concentrations present in the seed of the indicated treatments

Treatment	Total C (g 100 g^−1^)	Total N (g 100 g^−1^)	C/N ratio	Total crude protein (g 100 g^−1^)	Total soluble protein	Total soluble sugars	Starch
					(mg g^−1^ DW)	(mg g^−1^ DW)	(mg g^−1^ DW)
**(A)**							
Irrigation	3.7 ± 0.5	41.1 ± 0.1	11.2 ± 1.6	23.2 ± 13.6	18.4 ± 3.7	10.9 ± 1.6	199.2 ± 9.6
Water deficit	3.2 ± 0.2	40.7 ± 0.1	12.9 ± 0.9	19.7 ± 1.7	13.7 ± 2	15.2 ± 0.5	294.7 ± 11.4
	*P* < 0.05 (*)	*P* < 0.05 (*)	*P* < 0.005 (*)	*P* < 0.05 (*)	*P* < 0.05 (*)	*P* < 0.005 (***)	*P* < 0.005 (***)

Treatment	Total C (g/100 g)	Total N (g/100 g)	C/N ratio	Total Crude Protein (g/100 g)	Total soluble protein	Total soluble sugars	Starch
					(mg g^−1^ DW)	(mg g^−1^ DW)	(mg g^−1^ DW)
**(B)**							
Control	2.9 ± 0.4	39.8 ± 0.6	14.1 ± 1.8	17.9 ± 2.5	16.9 ± 5.2	11 ± 5	220.6 ± 59.5
Nodulated	4.1 ± 0.4	41.5 ± 0.2	10.1 ± 1	25.9 ± 2.4	21 ± 3.5	14.8 ± 3.1	259.5 ± 56.7
	*P* < 0.05 (*)	*P* < 0.05 (*)	*P* < 0.05 (*)	*P* < 0.05 (*)	*P* < 0.05 (*)	*P* < 0.05 (*)	NS

**Table 9:** Amino acid concentration (μmol g^−1^ DW) present in the seed of the indicated treatments

Treatment	GABA	Ala	Arg	Asn	Asp	Glu	Gln	His	Ile	Leu	Phe	Pro	Trp	Tyr	Val
**(A)**														
Irrigation	13 ± 3.7	9.3 ± 1.7	58.5 ± 15.8	94.3 ± 28.3	74 ± 8.1	139.1 ± 40.4	467.9 ± 25.3	2.6 ± 0.4	6 ± 1.2	6.6 ± 0.4	8.9 ± 0.9	61.7 ± 7.1	30.2 ± 8.5	12.9 ± 1	11 ± 1.2
Water deficit	10.1 ± 3.7	10.5 ± 1.3	48.8 ± 18.5	64.6 ± 32.9	62.5 ± 35.4	135.4 ± 58.4	400.6 ± 102.7	2.9 ± 1.1	5.1 ± 2	6.4 ± 1.9	9.9 ± 0.9	63.4 ± 5.9	67.4 ± 8	11.7 ± 1.2	9.1 ± 4.3
	*P* < 0.001 (***)			*P* < 0.01 (**)							*P* < 0.05 (*)		*P* < 0.001 (***)		

Treatment	GABA	Ala	Arg	Asn	Asp	Glu	Gln	His	Ile	Leu	Phe	Pro	Trp	Tyr	Val
**(B)**															
Control	8.3 ± 2.7	11 ± 3.9	34.8 ± 15.8	52.2 ± 16.1	52.728 ± 12.8	83.9 ± 33.8	489.2 ± 41.8	35.3 ± 7	3.4 ± 1	4.3 ± 1.6	7.7 ± 3.7	51.2 ± 14.7	59.7 ± 37.7	8.6 ± 0.7	5.5 ± 1.5
Nodulated	12.8 ± 4.8	17.1 ± 7.6	53.8 ± 17.1	118.3 ± 29.9	62.2 ± 7.7	180.2 ± 48.8	676 ± 200.3	4.2 ± 2.6	4.2 ± 1.3	7.2 ± 1.5	11.9 ± 3.8	66 ± 22	16.7 ± 12.9	12.5 ± 3.8	9.4 ± 2.2
	*P* < 0.01 (**)			*P* < 0.01 (**)	*P* < 0.01 (**)	*P* < 0.01 (**)	*P* < 0.05 (*)	*P* < 0.001 (***)		*P* < 0.05 (*)			*P* < 0.01 (**)	*P* < 0.05 (*)	*P* < 0.05 (*)

*Note*: Measures were done by analysis of ^1^H‐NMR vetch seed spectra observed in each sample.

**Table 10:** Sugar concentration (mmol g^−1^ DW) present in the seed of the indicated accessions

Treatment	Fructose	Galactose	Glucose	*myo*‐Inositol	Sucrose
**(A)**					
Irrigation	913.3 ± 92.3	32.8 ± 2.6	46.7 ± 2.9	88.8 ± 16	924.9 ± 112.5
Water deficit	965.6 ± 89.5	34.7 ± 12.7	46.7 ± 8.7	73.5 ± 16.1	1064.3 ± 97
					*P* < 0.01 (**)

Treatment	Fructose	Galactose	Glucose	*myo*‐Inositol	Sucrose
**(B)**					
Control	1287.6 ± 284.1	28.8 ± 11.6	51 ± 29.2	101.6 ± 15.2	956.1 ± 97.8
Nodulated	1055.5 ± 79.9	48.7 ± 39.8	91.9 ± 79.1	76.9 ± 15.8	1080.2 ± 154.6
	*P* < 0.05 (*)			*P* < 0.05 (*)	

Measurements were made by analysis of ^1^H‐NMR vetch seed spectra present in the seed under the indicated treatments.

**Table 11:** Total TCA organic acid content (mmol mg^−1^DW), present in the seed under the indicated treatments

Treatment	Acetate	Citrate	Formate	Lactate	Malate	Succinate	Choline	Trigonelline	Adenosine
**(A)**									
Irrigation	5 ± 0.6	294.6 ± 21.8	2.9 ± 0.5	12.2 ± 1.3	103.2 ± 7.5	5.9 ± 1.5	231 ± 19.7	17.2 ± 1.4	4.1 ± 1
Water deficit	5 ± 0.4	155.6 ± 48.5	3.1 ± 0.5	12 ± 0.7	84.8 ± 25.8	7 ± 0.4	220.3 ± 51.6	22.9 ± 3	4 ± 0.7
		*P* < 0.01 (**)						*P* < 0.01 (**)	

Treatment	Acetate	Citrate	Formate	Lactate	Malate	Succinate	Choline	Trigonelline	Adenosine
**(B)**									
Control	7.2 ± 2	165.5 ± 48.9	3.9 ± 0.2	15.7 ± 4.9	103.2 ± 41.4	10.2 ± 2.2	176.3 ± 27.7	29 ± 3.5	4 ± 2.7
Nodulated	8.4 ± 3.2	356 ± 54.8	4.4 ± 1	15 ± 2.4	161.8 ± 40	8.6 ± 1.1	236.9 ± 36.1	18.2 ± 2.3	6.7 ± 2.8
		*P* < 0.001 (***)			*P* < 0.01 (**)	*P* < 0.05 (*)	*P* < 0.05 (*)		*P* < 0.001 (***)

Measurements were made by analysis of ^1^H‐NMR vetch seed spectra observed in each sample.

**Table 12:** Total phenolic content, total flavonoid content, total tannin content, anthocyanins and DPPH activity at the indicated treatments

Treatment	TCP	Flavonoids	Tannins	DPPH	Anthocyanin
	(mg GAE g^−1^ DW)	mg RE g^−1^ DW	mg CE g^−1^ DW	μg TE g^−1^ DW	mg g^−1^ DW
**(A)**					
Irrigation	2.62 ± 0.49	9.07 ± 0.21	273.89 ± 29.14	9.93 ± 2.54	1.02 ± 0.08
Water deficit	2.63 ± 0.94	9.19 ± 0.64	273.63 ± 16.54	10.94 ± 4.08	0.92 ± 0.15
					*P* < 0.05 (*)

Treatment	TCP	Flavonoids	Tannins	DPPH	Anthocyanin
	(mg GAE g^−1^ DW)	mg RE g^−1^ DW	mg CE g^−1^ DW	μg TE g^−1^ DW	mg g^−1^ DW
**(B)**					
Control	2.94 ± 0.53	8.66 ± 0.59	246.73 ± 40.39	9.53 ± 3.04	0.76 ± 0.28
Nodulated	3.01 ± 0.63	9.14 ± 0.37	291.56 ± 34.82	9.23 ± 2.27	0.65 ± 0.22
		*P* < 0.05 (*)	*P* < 0.01 (**)		*P* < 0.05 (*)

Values are expressed per gram of seed dry weight (DW).

Abbreviations: CE, catechin equivalent; GAE, gallic acid equivalent; RE, rutin equivalent; TE, Trolox equivalent.

**Table 13:** Total mineral composition in different selected accessions on dried seeds under the indicated treatments

Treatment	Al	As	B	Ca	Cd	Co	Cr	Cu	Fe	K	Mg	Mn
	(mg kg^−1^)	(mg kg^−1^)	(mg kg^−1^)	(mg kg^−1^)	(mg kg^−1^)	(mg kg^−1^)	(mg kg^−1^)	(mg kg^−1^)	(mg kg^−1^)	(mg kg^−1^)	(mg kg^−1^)	(mg kg^−1^)
**(A)**												
Irrigation	4.27 ± 1.21	0.17 ± 0	5.3 ± 1.1	1.1 ± 0	0.02 ± 0.00	0.16 ± 0.09	0.28 ± 0.2	11.8 ± 1.1	84.9 ± 1.1	10.8 ± 0.5	1.2 ± 0.1	37.2 ± 1.7
Water deficit	11.65 ± 6.17	0.24 ± 0	5.6 ± 1.6	1.1 ± 0.1	0.02 ± 0.00	0.154 ± 0.05	1.5 ± 0.9	11.4 ± 0.6	91.6 ± 2.3	13.3 ± 0.2	1.4 ± 0.1	38.4 ± 2.2
									*P* < 0.05 (*)	*P* < 0.05 (*)		

Treatment	Mo	Na	Ni	Pb	P	Rb	Si	S	Sr	Ti	Zn
	(mg kg^−1^)	(mg kg^−1^)	(mg kg^−1^)	(mg kg^−1^)	(g kg^−1^)	(mg kg^−1^)	(mg kg^−1^)	(g kg^−1^)	(mg kg^−1^)	(mg kg^−1^)	(mg kg^−1^)
**(A)**											
Irrigation	42.65 ± 13.39	44.2 ± 24.1	0.5 ± 0.22	0.08 ± 0.1	4.25 ± 0.21	42.7 ± 1.9	15.2 ± 1.1	1.95 ± 0.21	4.23 ± 0.51	0.26 ± 0.03	60.6 ± 2.6
Water deficit	40.24 ± 12.87	96.7 ± 70	1.43 ± 0.37	0.21 ± 0.16	5.8 ± 0.14	49.7 ± 21.5	62.1 ± 20.2	1.9 ± 0.14	4.26 ± 0.46	1.44 ± 1.1	58.5 ± 0
	*P* < 0.05 (*)				*P* < 0.05 (*)						

Treatment	Al	As	B	Ca	Cd	Co	Cr	Cu	Fe	K	Mg	Mn
	(mg kg^−1^)	(mg kg^−1^)	(mg kg^−1^)	(g kg^−1^)	(mg kg^−1^)	(mg kg^−1^)	(mg kg^−1^)	(mg kg^−1^)	(mg kg^−1^)	(g kg^−1^)	(g kg^−1^)	(mg kg^−1^)
**(B)**												
Control	5.67 ± 2.55	0.06 ± 0.02	6.0 ± 0.3	1.2 ± 0.2	0.03 ± 0.01	0.30 ± 0.08	0.4 ± 0.2	10.1 ± 2.1	80.8 ± 18.7	11.9 ± 0.7	1.3 ± 0.1	39.3 ± 3.1
Nodulated	3.87 ± 1.21	0.17 ± 0.1	5.0 ± 1.2	1.0 ± 0.4	0.01 ± 0.01	0.16 ± 0.10	0.6 ± 0.5	6.9 ± 2.1	71.3 ± 8.3	10.9 ± 1	1.2 ± 0.1	33.5 ± 4.0
					*P* < 0.01 (**)	*P* < 0.01 (**)					*P* < 0.05 (*)	

Treatment	Mo	Na	Ni	Pb	P	Rb	Si	S	Sr	Ti	Zn
	(mg kg^−1^)	(mg kg^−1^)	(mg kg^−1^)	(mg kg^−1^)	(g kg^−1^)	(mg kg^−1^)	(mg kg^−1^)	(g kg^−1^)	(mg kg^−1^)	(mg kg^−1^)	(mg kg^−1^)
**(B)**											
Control	30.32 ± 13.35	36.7 ± 16.7	0.82 ± 0.59	0.19 ± 0.12	4.73 ± 0.7	83.7 ± 5.8	46.8 ± 29.2	1.97 ± 0.12	5.47 ± 1.05	1.11 ± 1.02	55.7 ± 7.6
Nodulated	30.28 ± 1.8	59.7 ± 74.4	0.78 ± 0.16	0.13 ± 0.08	3.77 ± 0.42	54.6 ± 6.5	14.8 ± 7	2.03 ± 0.06	4.27 ± 2.14	0.21 ± 0.06	52.6 ± 3.8
						*P* < 0.05					

*Note*: Measures were done by analysis of inductively coupled plasma optical emission spectrometry (ICS‐OES) vetch seed spectra observed in each sample. The content of Be, Bi, La, Li, Sb, Se, Tl, V was also measured with values under detection level (<0.01 mg Kg^−1^).

### Impact of rhizobial symbiosis on nutritional composition

Our data indicate that rhizobial symbiosis promotes an increase of total N content and proteins, both soluble and total crude proteins (Table 8(B)), with a significant specific increase in most of the amino acids, including GABA, Asn, Asp, Glu, Gln, Leu Tyr and Val, by at least 30%, and decrease in His and Trp (Table 9(B)). Regarding carbohydrate levels, a general rise in soluble sugars was observed in seeds from nodulated vetches (Table 8(B)), while fructose and *myo*‐inositol decreased (Table 10(B)).

The analysis of total phenolic components did not indicate relevant differences between nodulated or non‐nodulated plants. However, flavonoids and total tannins showed a significant increase in nodulated plants. A decrease in anthocyanins was also observed after this symbiotic treatment (Table 12(B)), together with an increase in citrate, malate, choline and adenosine, and a decrease in succinate (Table 11(B)). Evaluation of the mineral composition revealed a slight reduction in Mg and a significant decrease in the microelements Cd, Co and Rb in nodulated plants (Table 13(B)).

## DISCUSSION

One of the main purposes of the breeding process is the selection of productive varieties with good nutritional characteristics. This article explores the crop yield and nutritional differences among the grain of several vetch genotypes, which are crucial for selecting the most suitable variety for specific dietary and agricultural purposes. We emphasize that, in the present study, more than 80 parameters have been examined, including nine agro‐morphological traits of production and phenology, the analyses of 30 minerals, 15 amino acids, sugars, and other primary and secondary metabolites, in addition to the quantitative and qualitative determination of protein and carbohydrate composition. Our results indicate different nutritional profiles in the grain of 19 different *V. sativa* varieties representative of the CRF gene bank collection, with 545 genotyped accessions.^15^ These findings highlight that different landraces exhibit relevant nutritional characteristics compared with currently commercialized varieties.

One of the most valuable nutritional components of *V. sativa* grain is its high protein content. Different varieties of common vetch show considerable variation in protein levels, typically ranging from 25% to 30% of the grain dry weight.^10^, ^29^ Our studies show that the accessions analyzed present crude protein levels similar to those previously published, with BGE000529 and BGE001163 being those with the highest protein content. Moreover, the quality of the protein, measured by its amino acid composition, also differs among varieties. Thus, some varieties such as Aitana stand out for its high concentrations of several amino acids such as alanine, asparagine, glutamine, leucine and isoleucine. We also observed differences in the protein pattern of the grain extract among different genotypes, suggesting a specific enrichment of some proteins. Protein profiles may be altered due to modifications in the expression of genes involved in protein post‐translational modifications, synthesis and degradation. These changes also potentially affect the amino acid composition, altering the nutritional value of the seed. Our results also indicate significant differences in carbohydrate, sugar and starch composition. There is also a high diversity in terms of mineral content between different varieties, highlighting genotypes especially rich in Fe, Zn, Mg and other elements of nutritional interest, such as Verdor, BGE004356 and BGE004375. Similarly, the variability between genotypes is notable in terms of other primary and secondary metabolites. Our studies also indicate differences in antioxidant compounds (phenolic compounds, flavonoids, tannins and anthocyanins). Bioactive phenolic compounds present in legumes are involved in several physiological and metabolic processes, mainly related to their antioxidant activity. The main phenolic compounds present in legume seeds are phenolic acids, flavonoids and condensed tannins.^30^ Legumes have a wide range of total phenolic content, which is directly correlated with their antioxidant activity.^31^ Recent studies over six Greek vetch varieties have shown different metabolomics profiles that correlate with different genetic diversity.^32^ These data support the potential of genetic diversity for the selection of varieties with desirable nutritional characteristics, including high protein and carbohydrate content, and the presence of specific metabolites, minerals and antioxidants. Multivariate analysis by FAMD and HCPC revealed six distinct clusters, each showing unique phenological and biochemical profiles. For instance, cluster 2 (Verdor) stood out for its very early flowering and the highest seed‐to‐pod ratio – traits that could be particularly valuable for improving reproductive efficiency in environments with limited resources. In contrast, cluster 6 (Aitana) was remarkable for its extremely high phenolic content and antioxidant activity, suggesting potential for breeding programs focused on functional quality and stress resilience. Other clusters, such as cluster 4, combined early flowering with moderate biomass and seed weight, pointing to a balanced agronomic performance.

Legumes are known for their ability to establish a symbiotic relationship with rhizobial bacteria, stimulating the biological nitrogen fixation.^33^ The symbiosis process promotes modifications in important nitrogen metabolic pathways, including the biosynthesis of amino acids required for protein synthesis.^34^ Our results confirm that symbiosis in *V. sativa* promotes an increase of total N content and protein levels, both soluble and total crude proteins, and a rise in most of the amino acids. The amino acids with the greatest increases were Glu and Asn, which double their levels in nodulated plants. Remarkably, these two amino acids play an essential role as the main donors of amino group during amino acid biosynthesis, suggesting that they are key players in the regulation of this metabolic pathway also in common vetch grain.^35^ N is a critical nutrient for the synthesis of amino acids and proteins in plants. However, since control non‐inoculated plants are supplemented with NO_3_NH_4_ as N source, our results suggest that the crude N availability is not the only explanation for the observed higher levels. Probably, metabolic pathways involved in the synthesis of amino acids and proteins are influenced by rhizobial symbiosis, promoting a more favorable nutrient status for the legume. A general increase in total C content, carbohydrates and soluble sugars was also observed in seeds from symbiotic plants, probably due to an increase in photosynthetic activity. Consistent with this hypothesis, we also observed a dramatic increase in citrate and malate – key regulators of the carbon cycle and involved in the transport of energy and nutrients. Finally, we observed that nodulated plants have increased flavonoids, which play a crucial role in signaling and establishing the symbiotic relationship. Regarding mineral composition, only significant alterations were detected in the levels of Mg, Cd, Co and Rb. Recent research has demonstrated that the leaves of some nodulated legumes such as soybean and faba bean present an increase in Mg, P, K and Ca in leaves and a decrease in P and K concentrations in seeds.^18^ These results suggest that symbiosis with rhizobial bacteria not only influences nitrogen uptake by plants but also affects the absorption of other elements, promoting the production of seeds with distinct nutritional profiles compared to those that do not undergo this symbiotic interaction. In conclusion, our results indicate that symbiosis promotes important alterations in the nutritional components of vetch, due to modifications in the general primary metabolism of proteins, carbohydrates and other metabolic pathways, including photosynthesis and secondary metabolic pathways. Consequently, nodulated vetches produce seeds with improved nutritional profiles, including increased carbohydrate and protein levels.

Different abiotic stresses affect the yield and quality composition of grain legumes. Drought is one of the major environmental stresses that adversely affects plant growth and productivity. Rainfed legumes such as vetches are particularly sensitive to water scarcity. Drought directly affects crop growth and decreases grain yield. Moreover, recent studies have indicated that legumes subjected to different abiotic stresses produce seeds with altered nutritional profiles.^36^ Therefore, given the current climate change scenario, one of the challenges is to guarantee sufficient worldwide production and maintenance of the nutritional value of legumes as the most important source of vegetable proteins. Our results indicate that, in vetch under our severe drought conditions, a significant decrease in N content and total crude protein was observed, with specific alterations in some amino acids. Drought also promotes an increase in total soluble sugars, starch and sucrose. Interestingly, water deficit also promotes a significant increase in trigonelline in our assays. Trigonelline is an alkaloid, a product of niacin (vitamin B_3_) metabolism. that is frequently present in legume seeds and has various biological functions, including promoting drought resilience.^37^ Increased P, K, Fe and Mo were also observed. P and K are essential macronutrients to ensure the nutritional status of the plant under stress. Fe and Mo are micronutrients directly involved in photosynthesis and nitrogen fixation, respectively, and may enhance the physiological resilience after drought.^38^ In any case, understanding the mechanisms that regulate the effect of drought on nutritional composition is complex. Thus, in some pulses, mild drought increases grain protein content, but severe drought reduces grain protein content in addition to the levels of N, P, Fe and Zn.^36^ In soybean, drought promotes an increase in Ca, P, Cu, Mn, Mo and Zn, and a decrease of Na, K and Ca.^16^ These data suggest a complex nutritional regulation after drought, which may depend on the intensity and duration of stress, the tissue and the legume analyzed.

## CONCLUSIONS

Understanding the effect of genotype, drought and symbiosis on nutritional changes from an integrated perspective is essential for optimizing legume cultivation and ensuring the production of high‐quality, nutrient‐rich seeds. Notably, the wide range of amino acid content and other nutrients among different accessions is greater than the variations caused by environmental factors such as drought or rhizobial nodulation. This fact suggests the relevance of genetic diversity present in collections as a tool for nutritional breeding. Further research is necessary to explore the mechanisms underlying these improvements and to develop agricultural practices that maximize the benefits of rhizobial symbiosis, enable the selection of optimal varieties with desirable nutritional traits and diverse feed dietary requirements, and support the design of strategies to enhance grain quality and legume resilience to abiotic stresses, including drought. Estimating these effects is crucial for developing strategies to mitigate the impact of drought on legume crops, and guarantee the availability of high‐quality, nutritious seeds.

## AUTHOR CONTRIBUTIONS

LDR and ERP: conceptualization, funding acquisition, resources and supervision. MLR, CCH and ERP: greenhouse assays. TM and LDR: phenotypic field trials. ERP: formal analysis, data visualization and project administration. LDR and ERP wrote and edited the paper. All authors contributed to the article, revised and approved the submitted version.

## FUNDING INFORMATION

This work was supported by grants PDI2021‐122138OR‐I00, from the Spanish Ministerio de Ciencia e Innovacion and Agencia Estatal de Investigacion of Spain (MCIN/AEI/10.13039/501100011033/FEDER; UE) and by the 'Severo Ochoa Program for Centres of Excellence in R&D' (Agencia Estatal de Investigación of Spain, grant CEX‐2020‐000999‐S to the CBGP). CCH is supported by PRE2022‐104860, funded by MCIN/AEI. CSIC partially supports open‐access publication fees.

## CONFLICTS OF INTEREST

The authors declare no conflicts of interest.

## Supporting information


**Figure S1.** Diagram and timeline of the procedures performed for inoculation (A) and drought (B) treatments. Detailed information is included in the Materials and Methods section.

**Table S1.** Accession numbers and passport data of the common vetch varieties that have been analyzed in this work.

**Table S2.** Specific values of measurements of agro‐morphological traits for selected accessions of common vetch (*V. sativa*).

**Table S3.** Table of the StatAdvisor component weights for the different parameters, showing the main contributions.

## Data Availability

Details regarding the data supporting reported results can be found in supplementary material and in the publicly CRF repository https://eurisco.ipk-gatersleben.de/apex/eurisco/r/eurisco/home.
