# Supplementary material for: Influence of genetic diversity, drought stress and rhizobial symbiosis on the nutritional quality of common vetch (Vicia sativa L.) grain
Source: J Sci Food Agric. 2026 Mar 2;106(7):3952–70. doi: 10.1002/jsfa.70410 (PMC13067090; doi:10.1002/jsfa.70410)
Supplement: Supplementary file 1 — Figure S1. Diagram and timeline of the procedures performed for inoculation (A) and drought (B) treatments. Detailed information is included in the Materials and Methods section. [file JSFA-106-3952-s004.pdf]

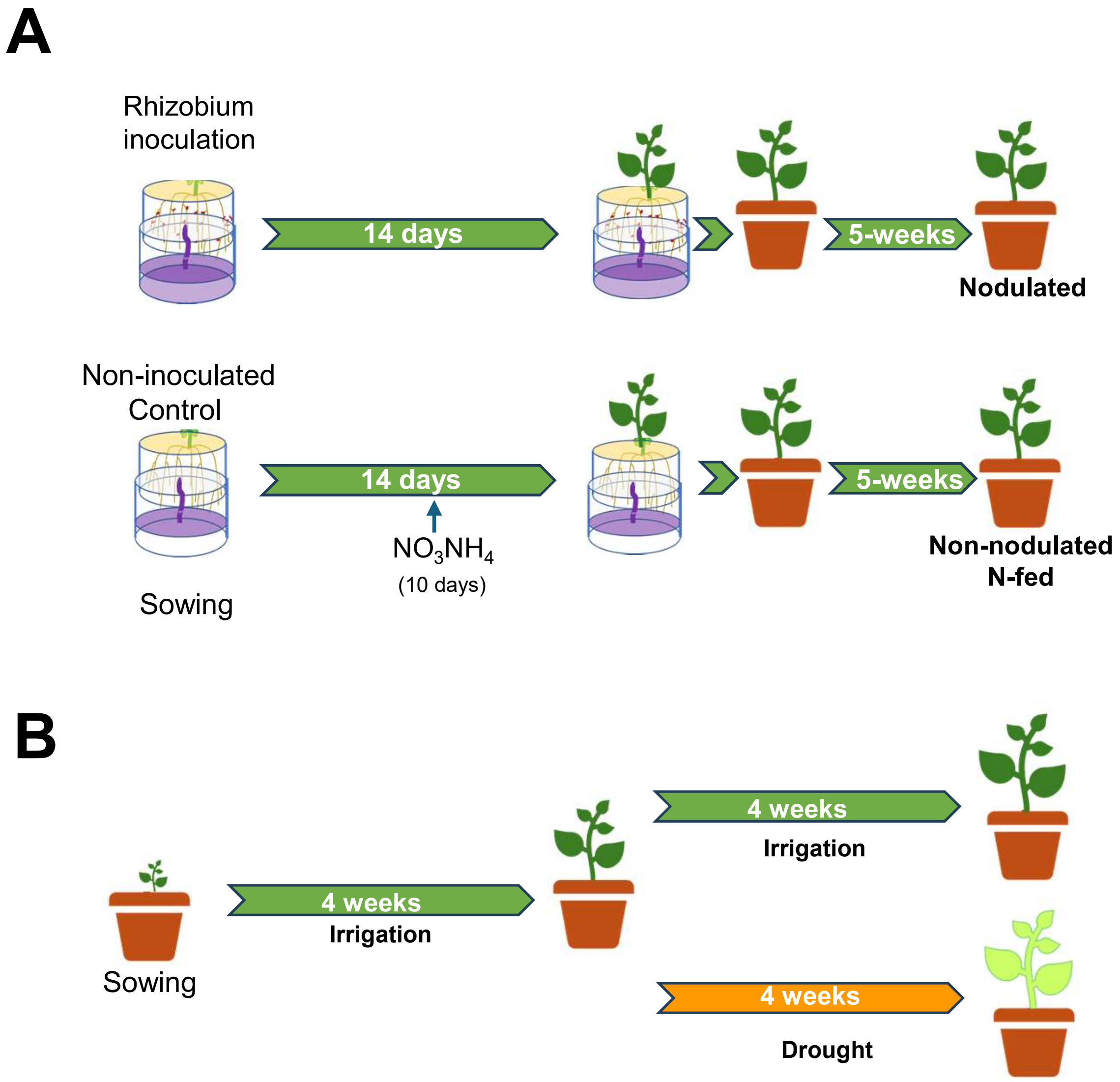

Figure S1. Diagram and timeline of the procedures performed for inoculation **(A)** and drought **(B)** treatments. Detail information is included in the materials and methods section.
